# Multiscale Manufacturing of Recyclable Polyimide Composite Aerogels

**DOI:** 10.1002/adma.202411599

**Published:** 2024-11-13

**Authors:** Mengmeng Li, Tingting Wu, Zhiyang Zhao, Lei Li, Tongxin Shan, Hui Wu, Robert Zboray, Francesco Bernasconi, Yongjie Cui, Peiying Hu, Wim J. Malfait, Qinghua Zhang, Shanyu Zhao

**Affiliations:** ^1^ Laboratory for Building Energy Materials and Components Swiss Federal Laboratories for Materials Science and Technology Empa, Überlandstrasse 129 Dübendorf 8600 Switzerland; ^2^ College of Materials Science and Engineering Donghua University Shanghai 201620 P. R. China; ^3^ Institute of Sustainability for Chemicals Energy and Environment (ISCE2) Agency for Science Technology and Research (A*STAR) 1 Pesek Road Jurong Island 627833 Singapore; ^4^ State Key Laboratory of New Ceramics and Fine Processing School of Materials Science and Engineering Tsinghua University Beijing 100084 China; ^5^ National Engineering Research Center of Electric Vehicles Beijing Institute of Technology Beijing Beijing 100081 China; ^6^ Center for X‐ray Analytics Swiss Federal Laboratories for Materials Science and Technology Empa Dübendorf 8600 Switzerland; ^7^ Materials for Energy Conversion Laboratory Swiss Federal Laboratories for Materials Science and Technology Empa Dübendorf 8600 Switzerland; ^8^ Department of Materials ETH Zürich Zürich 8093 Switzerland; ^9^ School of Energy and Materials Shanghai Polytechnic University Shanghai 201209 China

**Keywords:** aerogel, density functional theory, high‐temperature resistance, multi‐scale manufacturing, recyclability

## Abstract

Mitigating embodied emissions is becoming increasingly crucial as the energy supply shifts toward more sustainable sources. Bio‐based materials present a potentially more sustainable alternative to synthetic polymers; however, it often do not yet match the performance of synthetic materials. Given the ongoing reliance on high‐performance, high‐environmental‐impact materials, it is essential to ensure their complete recyclability. Aerogels, recognized by IUPAC as one of the top ten emerging technologies, are witnessing rapid market growth in thermal insulation and thermal protection applications. In certain applications, synthetic and composite aerogels exhibit superior performance, particularly under high temperatures. Here, molecular simulation tools are employed to elucidate the interaction forces between polymers and solvents, develop a recycling strategy for polyimide‐based aerogels, and demonstrate their application in thermal protection for firefighter textiles and thermal runaway protection for Li‐ion battery packs. These composites are engineered for disassembly, allowing for the complete recovery of starting materials without any degradation of components after multiple recycling cycles. The recyclable composites can be fabricated using various manufacturing techniques to produce fibers (1D), membranes (2D), and complex structures (3D). This unique combination of outstanding performance and excellent recyclability facilitates the sustainable utilization of aerogels in protective clothing, electric mobility, consumer goods, and aeronautics.

## Graphical abstract

Mitigating embodied emissions is becoming increasingly crucial as the energy supply shifts toward more sustainable sources. A recycling strategy is guided by molecular simulation to reach recycling of high‐performance polyimide‐based aerogels. The recyclable composites can be fabricated using various manufacturing techniques to produce fibers (1D), membranes (2D), and complex structures (3D). The unique combination facilitates the sustainable utilization of outstanding aerogel composites in protective clothing, electric mobility, consumer goods, and aeronautics.

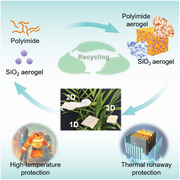

## 1 Introduction

Composite aerogels derived from high‐performance polymers preserve the exceptionally lightweight and insulating characteristics of traditional aerogels^[^ ^1^, ^2^ ^]^ whilst harnessing the advantages of engineering polymers, including elasticity, high mechanical strength, and surface functional versatility. Polyimide stands out among polymers due to its exceptional thermal stability, mechanical toughness, and chemical resistance, with extensive applications in microelectronics,^[^ ^3^ ^]^ gas separation,^[^ ^4^ ^]^ and energy storage.^[^ ^5^ ^]^ Polyimide aerogels, with their low density and high porosity, open up new applications ranging from high‐temperature thermal protection^[^ ^6^ ^]^ to sound‐absorbing materials^[^ ^7^ ^]^ and thermal infrared materials.^[^ ^8^ ^]^ In addition, the combination of polyimide with materials such as graphene,^[^ ^9^ ^]^ MXene, metal oxide nanoparticles,^[^ ^10^ ^]^ and silica aerogel,^[^ ^11^ ^]^ has unlocked new functionalities and uses.

The performance advantages of polyimide composite aerogels are offset by significant challenges in terms of environmentalimpact, recyclability, and end‐of‐life solutions. Aromatic polyimide itself is not readily recyclable due to the insolubility and infusibility resulting from its stable chemical structure and highly conjugated aggregated state.^[^ ^12^, ^13^ ^]^ Additionally, the complexity of composite structures increases the difficulty of separating the polymer matrix from the compounding materials.

In the context of aerogel recycling and sustainability, there is a dearth of studies on composite materials. The focus has primarily been on pristine polymer aerogels, with three main approaches: the utilization of bio‐based raw materials,^[^ ^14^ ^]^ exploring reuse applications,^[^ ^15^ ^]^ and developing recyclable materials.^[^ ^16^, ^17^, ^18^, ^19^ ^]^ However, the current practice of reusing these materials follows a linear economy model on single‐component materials, where products are eventually discarded at the end of their lifecycle, resulting in a loss of feedstock value. In stark contrast, the development of fully recyclable high‐performance composite aerogels for high‐added‐value applications presents an opportunity to establish a sustainable circular economy that reduces waste, repurposes materials, and minimizes their environmental footprint.

We present a novel polyimide‐based aerogel, incorporating silica aerogel as a filler, to develop design‐for‐disassembly composite aerogels. Polyimides with rigid and conjugated molecular structures possess strong intermolecular forces, complicating their reuse after processing and molding. However, incorporating flexible or bulky side groups in the polyimide can reduce these interactions and enhance solubility. Guided by simulations, the selected polyimide, with the BTDA@TDI_MDI structure, exhibits good solubility in DMAc, enabling the recycling of these innovative multiscale composite aerogels multiscale after processing. The resulting composite aerogels demonstrate excellent mechanical properties and thermal stability, indicating significant potential for thermal protection fabrics and lithium‐ion thermal runaway protection.

## 2 Result and Discussion

### 2.1 1D, 2D, and 3D Fabrication of Polyimide‐Silica Composite Aerogels

**Figure 1:**
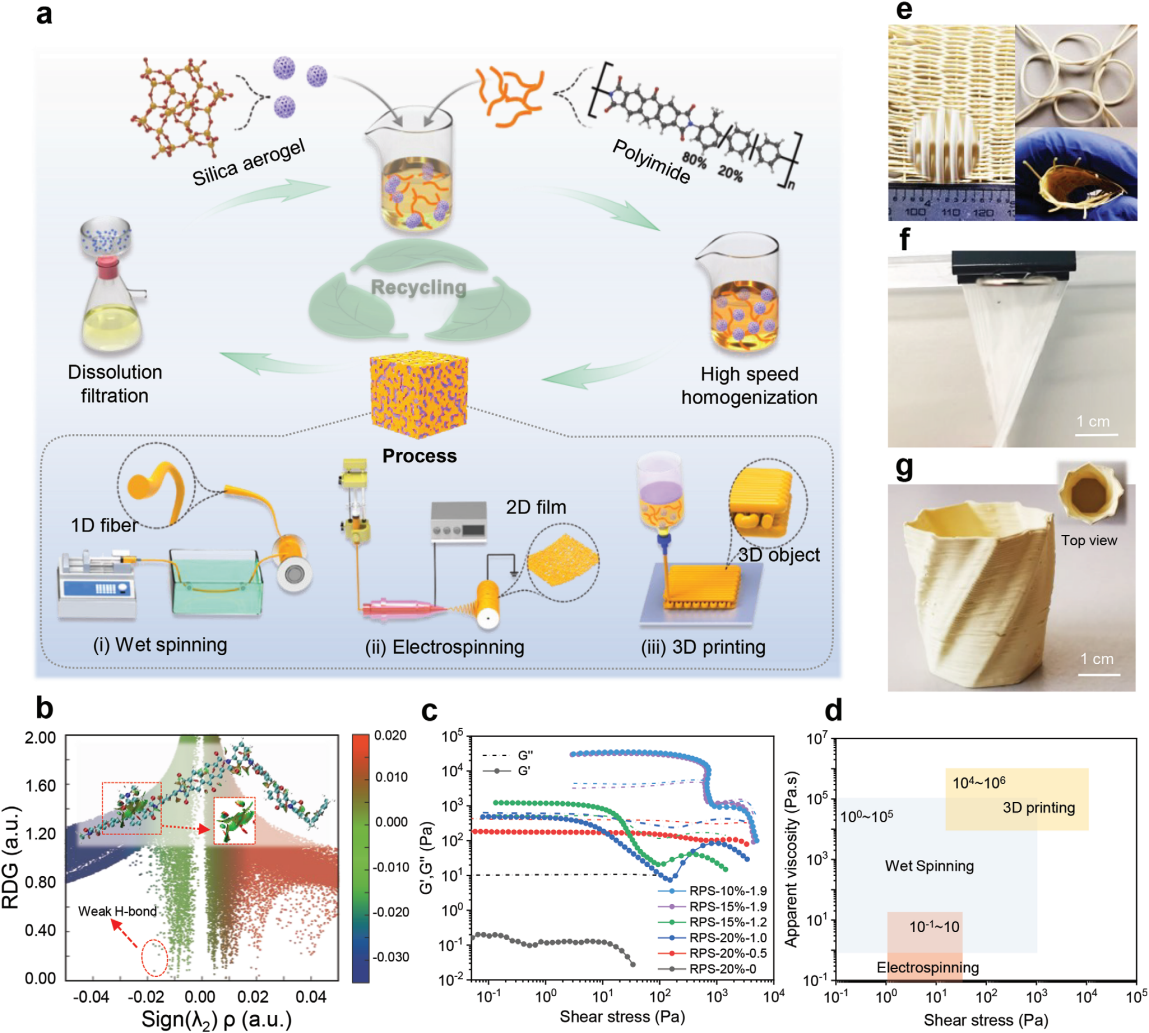
Synthesis of recyclable Polyimide‐silica aerogel composites. a) Fabrication and recycling scheme of polyimide‐silica aerogels with multiscale morphology. b) Schematic diagram of the interaction between polyimide and DMAc and the integration results of RDG. c) Storage (G′) and loss (G″) modulus versus shear stress of polyimide‐silica dispersions with different filler contents. d) Viscosity and shear stress range suitable for electrospinning, wet spinning, and 3D printing. Pictures of e) 1D fibers produced by wet spinning, f) 2D film produced by electrospinning, and g) 3D printed twisty vase.

In the pursuit of recyclable polyimide composite aerogels, maintaining the initial properties of the components during polymer recycling is crucial. To understand the polymer behavior in composite preparation, the mutual interaction forces of the polymers in solvents were simulated using density functional theory (DFT) (Figure S1a, Supporting Information), to assess the solubility of the selected structured polymer in various solvents. As for the highly conjugated polyimide, we figured out similar extreme values of the molecular surface electrostatic potential (ESP_min_ and ESP_max_) (Figure S1b, Supporting Information) and molecular polarity index (MPI) (Figure S1c, Supporting Information) to the solvent are prone to a good solubility. A flexible and large free‐volume molecular chains (co‐polyimide of 3,3′,4,4′‐benzophenone tetracarboxylic dianhydride, methylphenylenediamine, and methylenediamine, BTDA@TDI_MDI) shows a good match of ESP and MPI to the typical polyimide solvent dimethylacetamide (DMAc), compared to Upilex and Kapton. The integration results of reduced density gradient (RDG) indicate that the interaction between BTDA@TDI_MDI molecules and DMAc is primarily Van der Waals forces and weak hydrogen bonds (**Figure** **1**b; Figure S1d, Supporting Information), promoting the formation of stable solutions. Following, we use the recyclable polyimide‐silica (RPS) composite aerogel for the demonstration, the complete dissolution of the BTDA@TDI_MDI particles in DMAc was prepared, and silica aerogel particles (5–20 µm, Cabot Aerogel) can be introduced as a high‐performance filler phase and concurrently acting as a rheology modifier (Figure 1a; Figure S2, Supporting Information). Stable composite aerogels were obtained and are denoted as RPS‐x%‐y, indicating x wt.% polyimide in the final formulation and loaded with y grams of silica aerogel powders, while maintaining a constant total mass of 20 g of the polyimide solution, i.e., ≈20 mL (Table S1, Supporting Information).

The apparent sol viscosity can be tuned from 10^2^ to 10^6^ Pa·s by adjusting silica aerogel (0–8.8 wt.%) and polyimide concentration (10–20 wt.%) (Figure S2, Supporting Information). All composite formulations exhibit shear‐thinning behavior with higher apparent viscosities, higher yield stress, and more pronounced shear‐thinning for higher silica aerogel loadings (Figure 1c; Figure S2, Supporting Information). The tunable rheology enables adaptability to a variety of fabrication technologies such as wet spinning (1D fibers, Figure 1e), electrospinning (2D film, Figure 1f), and additive manufacturing (3D complex objects, Figure 1g). Wet spinning, the process of injecting precursor solutions directly into a coagulation bath, exhibits more lenient viscosity requirements, ranging from 10^0^–10^5^ Pa·s.^[^ ^20^, ^21^ ^]^ Electrospinning, on the other hand, necessitates a fine‐tuned, low viscosity of 10^0^–2 × 10^2^ Pa·s to create morphologically intact nanofiber membranes.^[^ ^22^, ^23^ ^]^ Additionally, the filler particles should be at least four times smaller than the nozzle. In contrast, 3D printing requires a highly viscous formulation with finely controlled flow properties during shear, and the printed filament should exhibit sufficient yield stress after extrusion to be self‐standing, necessitating a high apparent viscosity of 10^4^–10^6[^ ^24^, ^25^ ^]^ and fast rebound after extrusion (Figure 1d).

**Figure 2:**
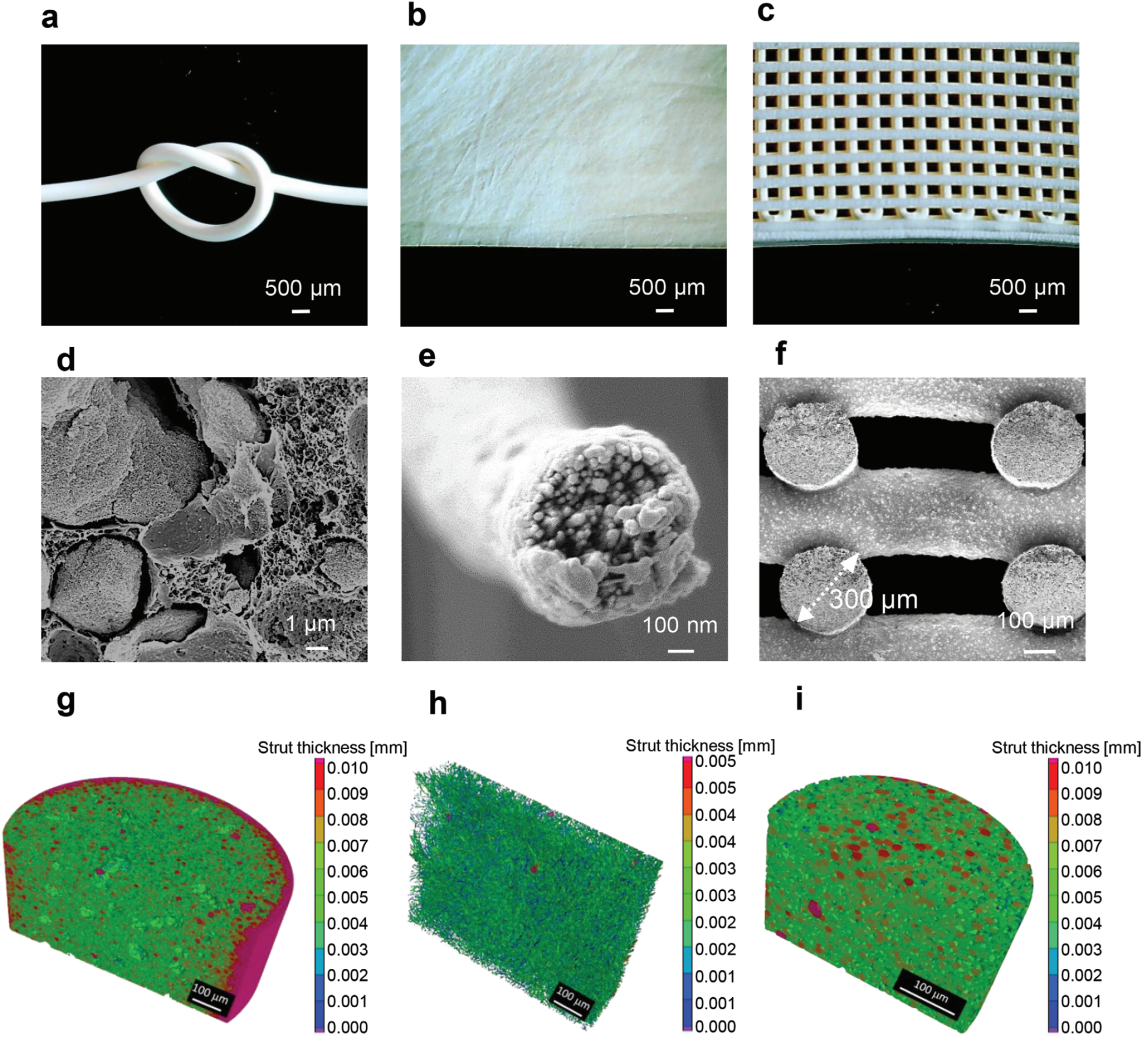
Microstructures of RPS composites prepared by wet spinning, electrospinning, and direct‐ink‐writing. Microphotograph of a) RPS‐20%−1.0 aerogel fiber, b) RPS‐20%−0.5 electrospun nanofiber film, and c) RPS‐15%−1.9 printed cube. Cross‐section SEM images of d) aerogel fiber, e) nanofiber in the electrospun film and f) the printed cube. Cross‐section and tomographic rendering (0.4^3^–0.9^3^µm^3^, effective pixel size of 18 µm) of g) aerogel fiber, h) electrospun nanofiber film, and i) printed cube.

During the wet spinning of 1D fibers, a thread of RPS sol (in DMAc) is extruded through an antisolvent ethanol coagulation bath to induce rapid phase separation and gelation to form a composite wet gel fiber. Since gelation occurs almost immediately after extrusion, the rheological requirements are relatively wide (Figure 1d), but a sol with intermediate silica aerogel content (RPS‐20%−1.0) was selected considering as a compromise between fiber flexibility and tensile strength and thermal insulation performance. A series of aerogel fibers with diameters ranging from 231 to 780 µm were spun by varying the nozzle size (Figure S3a, Supporting Information). After supercritical CO_2_ drying (SCD), the composite aerogel fibers display a circular cross‐section with diameters close to the nozzle size (e.g., 785 µm for an 810 µm nozzle, **Figure** **2**a; Figure S3d, Supporting Information), indicating low shrinkage during the spinning and drying processes. Silica aerogel particles uniformly dispersed in the porous polyimide skeleton, forming the desired aerogel‐in‐aerogel composite structure (Figure 2d). The fiber surfaces are denser with smaller pores, caused by fast gelation from the coagulation bath or/and a skin effect during drying (Figure 2g; Figure S3g, Supporting Information). At intermediate silica aerogel contents, the fibers are sufficiently supple to enable weaving into a flexible textile (Figure 1e).

Because electrospinning requires low viscosity (Figure 1d), and the addition of silica aerogel particles increases viscosity, a formulation with low silica aerogel content (RPS‐20%−0.5) was selected. Nanofibers were spun with a multi‐jet electrospinning system using 10 kV accelerating voltage, ≈8 cm needle‐collector distance for 2 h, resulting in a nanofiber film with high flexibility and strong potential in flexible wearable devices (Figures 1f and 2b; Figure S3b, Supporting Information). In contrast to wet spun fibers, electrospinning produces superfine fibers of ≈700 nm in diameter (Figure 2e; Figure S3e, Supporting Information). Although the silica aerogel particles (average 20 µm) are smaller than the nozzle size (210 µm), they are much too large to be incorporated throughout the electrospun nanofibers, but they are locally present in the form of polyimide‐wrapped silica aerogel particles (Figure 2h; Figure S3e,h, Supporting Information). Surprisingly, even though the electrospun fiber is dried during the spinning process, the inner fiber structure remains porous, possibly because the very fast drying of small‐diameter fibers limits pore collapse during evaporative drying (Figure 2e).

Direct ink writing was applied to produce complex 3D objects.^[^ ^26^, ^27^ ^]^ Relatively high silica aerogel loadings (RPS‐15%−1.9) were selected to reach a sufficiently high viscosity and printing fidelity. Customized shapes with fidelity were successfully printed (Figures 1g and 2c; Figure S4 and Movie S1, Supporting Information) over a range of nozzle pressures (Figure S5, Supporting Information). After printing, the wet gel objects were immersed in an anti‐solvent ethanol bath and finally dried using SCD. The diameter of the printed filaments is ≈300 µm (Figure 2f), consistent with the nozzle size of 410 µm, indicating very little shrinkage during the printing, gelation, and drying processes, guaranteeing the high fidelity of the final objects. In its inner morphology, the polyimide displays an aerogel‐in‐aerogel structure (Figure 2i; Figure S3f, Supporting Information) similar to that of the wet‐spun fibers (Figures 2d,g). On the surface, the printed filaments do not have a pronounced denser skin, but a more open polymer structure that wraps the (slightly protruding) silica aerogel particles (Figure S3i, Supporting Information).

**Figure 3:**
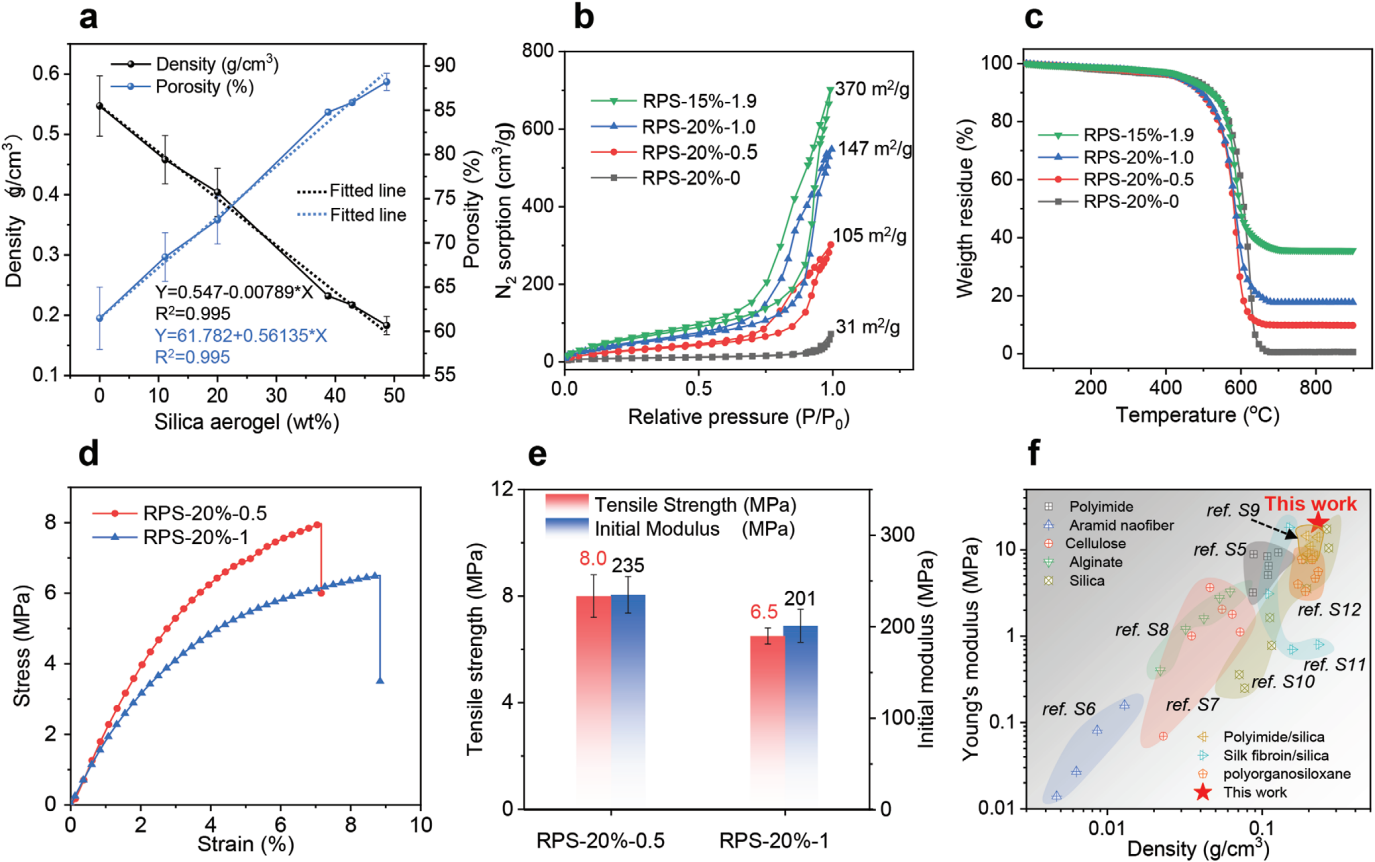
Physical properties of the RPS composite aerogels. a) Density and porosity of RPS composite aerogels with different silica aerogel contents. b) N_2_ sorption isotherms and c) TGA curves of RPS composite aerogels. d) Stress‐strain curves of RPS aerogel fibers. e) Tensile strength and initial modulus of RPS aerogel fibers. f) Comparison of the aerogels in terms of density and compressional Young's modulus (the detailed data is listed in Table S2, Supporting Information).

Aside from modulating the sol rheology, silica aerogel plays a crucial role in enhancing the pore structure and related thermal stability of the composite aerogels. Composite aerogel monoliths were prepared via sol casting to facilitate the measurement of density, porosity shrinkage, and thermal conductivity as a function of sol formulation. When the silica aerogel concentration increases, the density of the composite aerogel decreases from 0.547 to 0.183 g cm^−3^, and porosity increases from 62% to 89%. The density and porosity of the prepared composite aerogels vary linearly with increasing silica aerogel content, despite variations in polyimide concentration, indicating the importance of the silica aerogel filler (**Figure** **3**a). The specific surface area increases from 31 to 370 m^2^ g^−1^ as the silica aerogel content increases from 0 to 1.9 g in total 20 mL gels (Figure 3b). Thanks to the excellent thermal stability of both silica aerogel and polyimide, the composite aerogels display a high thermal decomposition temperature of 583 °C in the air (Figure 3c) and the residual mass at 900 °C gradually increased with the increase of silica content, up to 35.5% for RPS‐15%−1.9.

Brittleness and dust release continue to be a significant obstacle to the widespread application of aerogels, particularly for silica aerogels. The RPS composite aerogels obtained in this study inherit the robust characteristics of polyimide. The RPS composite aerogels exhibit impressive strength‐to‐weight ratios, even in response to tensile and bending forces. The 1D wet‐spun fiber (RPS‐20%−1.0) can withstand a weight of 50 grams without breaking, demonstrating good flexibility and mechanical properties (Figure S6a, Supporting Information). The 2D film (RPS‐20%−0.5), with a thickness of ≈50 µm, is flexible enough to be twisted or folded or support a magnet weighing 9.4 g (Figure S6b, Supporting Information). The 3D honeycomb is light enough to be supported by a flower's petal tips (Figure S3c, Supporting Information) but can maintain an intact morphology under >50 000 times its weight (Figure S6c, Supporting Information), e.g. an adult human (Figure S6d and Movie S2, Supporting Information). In more detail, the wet‐spun composite aerogel fibers (RPS‐20%−0.5 and RPS‐20%−1.0) exhibit typical plastic deformation during tensile testing, with elongations of 7.2% and 8.8%, tensile strengths of 8.0 and 6.5 MPa and initial moduli of 235 and 201 MPa, respectively (Figures 3d,e). These values surpass those reported aerogel fibers within a similar density range.^[^ ^2^, ^28^, ^29^, ^30^ ^]^ Compared to commercial, non‐porous, high‐performance fibers, the RPS aerogel fibers exhibit lower mechanical properties, but provide significant advantages in terms of lower density and a higher decomposition temperature, making them highly promising for applications in lightweight thermal insulation fabrics (Figure S6e and Table S3, Supporting Information). The tensile strength decreases with increasing silica aerogel content due to the lower density and brittle nature of silica aerogels, but, somewhat surprisingly, the elongation at break increases. The content of silica aerogel influences the mechanical properties of the composite aerogels. For the composite aerogel fibers for example, tensile strength and initial modulus decrease with increasing silica aerogel content due to the inherent brittleness of silica aerogel. In contrast, the maximum elongation first increases and then decreases rapidly upon silica aerogel addition (Figures S7a,b, Supporting Information). We hypothesize that this initial increase is due to the increased homogeneity of the microstructure by reducing the formation of larger, finger‐like pores (Figure S7c, Supporting Information), which in turn enables a more uniform stress distribution, and thus improves flexibility and elongation. At the highest silica aerogel loadings, defects gradually appear on the surface of the fibers (Figure S7c, Supporting Information), which also leads to a further decrease in the mechanical properties of the fibers. The high‐temperature dimensional stability of the composite aerogel fibers improved with increasing silica aerogel content and there may be no upper limit for silica aerogel loading to enhance shrinkage resistance (Figure S8, Supporting Information). However, the inferior mechanical properties of silica aerogel resulted in a corresponding decline in the mechanical performance of the fibers at high silica aerogel loadings. Generally, RPS‐20%−1.0 is the preferred formulation for the preparation of composite aerogel fibers, as it displays good thermal stability, high tensile strength, initial modulus, and high elongation.

Meanwhile, the aerogel fibers were prepared using DMAc and NMP as the solvents showed similar chemical structures and microstructures, resulting in no significant differences in their performance (Figures S9a–d, Supporting Information). However, DMAc offers a shorter dissolution time, facilitating more efficient preparation of the composite aerogel (Figure S9e, Supporting Information).

The RPS‐15%−1.9 composite aerogel monolith has a high compressional Young's modulus and low density compared with previously reported aerogels, as long as low thermal conductivity of 23.7 mW/(m.K) (Figure 3f; Figure S10, Supporting Information), highlighting their potential for applications in lightweight thermal insulation fields. In summary, the RPS composites, with their aerogel‐in‐aerogel structure, successfully retain the lightweight, superinsulation characteristics of silica aerogel and the excellent mechanical properties of polyimide.

### 2.2 Polyimide‐Silica Composite Aerogel Recycling

**Figure 4:**
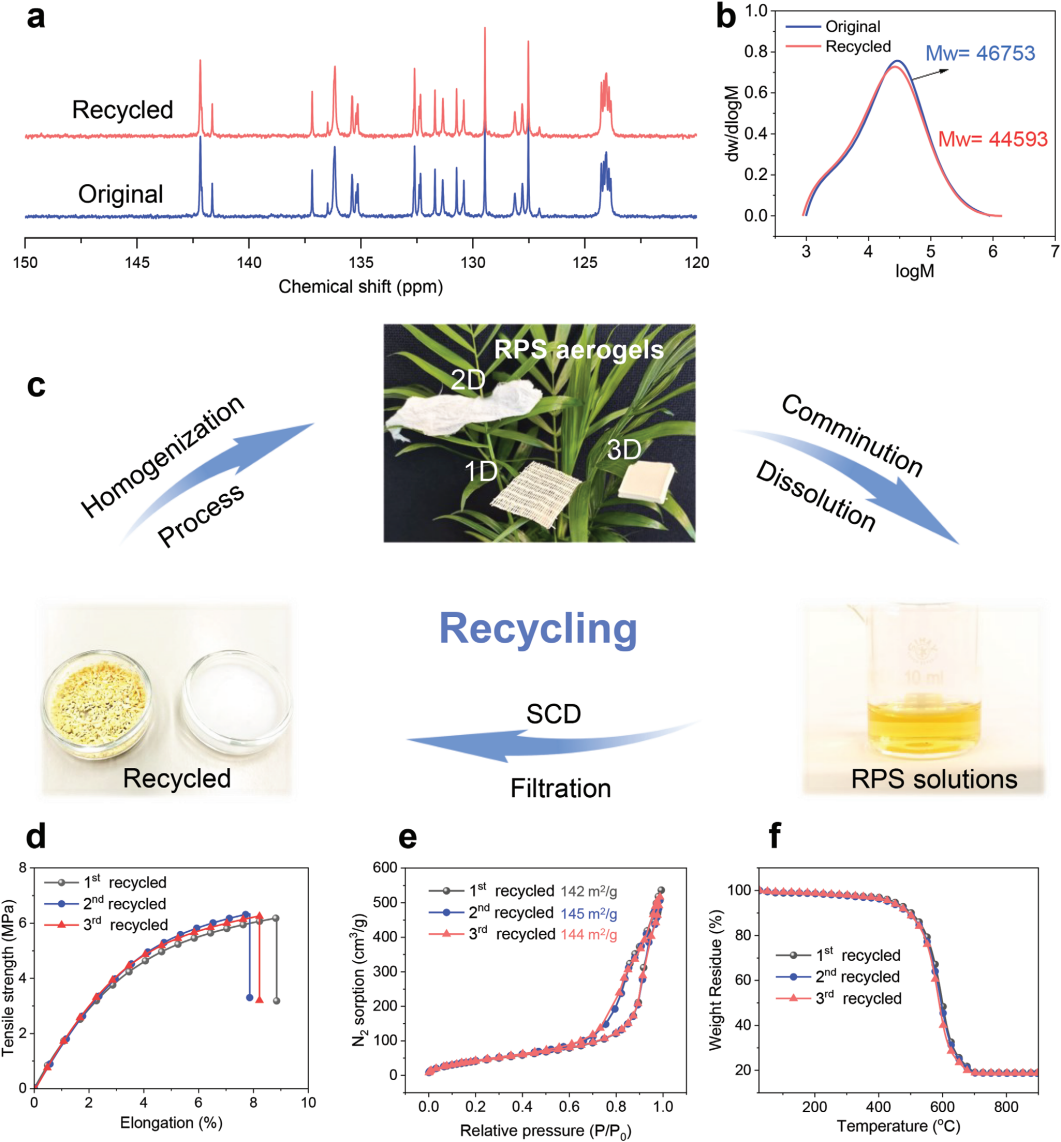
Recycling of RPS composite aerogel. a) ^13^C NMR spectra, b) molecular weight and distribution, c) Recycling processes of RPS composite aerogels, d) stress‐strain curves, e) N_2_ sorption isotherms, and f) TGA curves of the recycled RPS composite aerogels.

The flexible and large free‐volume molecular chains of polyimide render it soluble in polar non‐protonic solvents, such as DMAc, NMP, DMSO, and DMF (Figure 1b Figure S11g, Supporting Information). At the same time, the inert nature of silica aerogel enables a clear separation and easy recovery of the silica after the dissolution of polyimide. Consequently, the RPS composite aerogels demonstrate excellent recyclability. Following a straightforward polymer dissolution, silica filtration, and drying process, the polyimide and silica aerogel can be recovered separately. Spectroscopic (FTIR‐ATR and NMR) and chromatographic analysis reveal that the recycled polyimide maintains its original chemical structure (**Figure** **4**a; Figures S11a–c, Supporting Information), molecular weight (4.5 × 10^4^–4.7 × 10^4^) and polydispersity (4.5–4.8) (Figure 4b) with a total recycling yield >98%. The silica aerogel also preserves its original chemical and pore structure after recycling (Figures S11a,d–f, Supporting Information). The specific surface area (≈723 m^2^ g^−1^) and pore diameter (≈20 nm) of the silica aerogel powder are maintained after the recycling processes, with a recycling yield of 85%. The loss is primarily attributed to lab‐scale handling losses (filtration and drying). New composite aerogel fibers were produced from the recovered polyimide and silica aerogel by wet spinning to identify a possible degradation in performance (Figure 4c). Recycled RPS‐20%−1.0 aerogel fibers display a tensile strength of 6.2–6.3 MPa and elongation of 7.8–8.8% (Figure 4d), comparable to the original samples. The nitrogen sorption isotherms, specific surface area, and pore structure also remain unchanged (Figure 4e; Figure S12, Supporting Information) and the same holds for the thermal stability (Figure 4f). In summary, RPS composites can be separated easily into their initial components, with high yield, and without chemical or structural degradation. These components can then be reused for other applications, including the preparation of new RPS composites that are indistinguishable from those prepared from primary raw materials. Additionally, this method can be applied to prepare other high‐performance composites to facilitate efficient component separation and reuse. For example, CNT@Polyimide composite aerogel fibers prepared through this novel method allow for the effective separation of CNT and polyimide from their composite (Figure S13, Supporting Information).

### 2.3 Recyclable Polyimide‐Silica Composite Aerogels for High‐Temperature Shielding

The recyclable polyimide‐silica aerogel composites exhibit excellent thermal stability, thermal insulation, and mechanical performance that silica, polyimide, and their composite aerogels are renowned for thermal protection applications. Polyimide‐silica aerogel composites are not only chemically stable up to high temperature, but also have excellent dimensional stability during extended high‐temperature exposure.^[^ ^31^ ^]^ For example, the RPS‐20%−1.0 composite aerogel fibers maintain their chemical structure and microstructure even when heated at 200 °C for 1 h under an air atmosphere (Figures S14a–c, Supporting Information). What's more, the thermal stability and performance of RPS‐20%−1.0 composite aerogel can resist temperatures up to 500 °C, with stable and outstanding heat shielding performance and high thermal stability, i.e., the composite maintains good chemical and physical properties after exposure (Figures S14d,e, Supporting Information). Also, a 3D‐printed composite aerogel honeycomb (RPS‐20%−1.9), when placed on a hot plate at 300 °C, exhibited a surface temperature of only 35 °C (Figure S15a, Supporting Information) and maintained its shape and did not burn after exposure to a flame (30 s at ≈750 °C) (Movie S3 and Figures S15b,c, Supporting Information).

**Figure 5:**
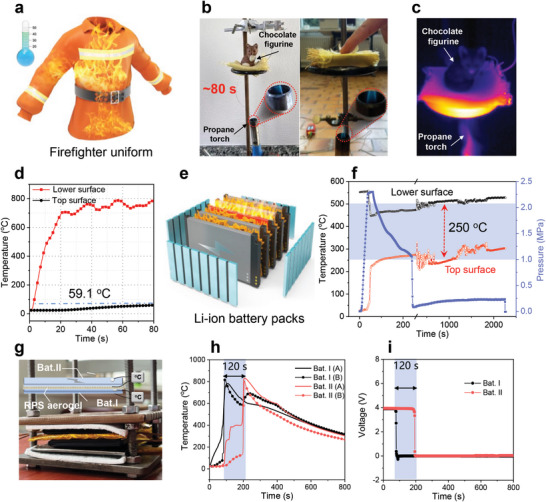
High‐temperature shielding of the RPS aerogels. a) Potential application of RPS aerogels in firefighting uniform. b) Thermal protection and fireproof property of RPS aerogel textile. c) Infrared thermal images and d) Real‐time temperature changes on the bottom and top surfaces of the textiles during the thermal protection test. e) The application of RPS aerogels in Li‐ion battery packs under thermal runaway protection and the structure diagram. f) Real‐time temperature and pressure variation curves of the composite aerogel during the standard battery thermal failure test. g) Photo of the thermal test of the Li‐ion batteries under the protection of RPS aerogel textile, h) real‐time temperature curves, and i) voltage of the Li‐ion batteries.

Both 1D and 2D polyimide‐silica composites are suitable for textile production, making them a viable option for specialized clothing that demands resistance to external heating, such as fire‐fighter uniforms (**Figure** **5**a). As a proof‐of‐concept, a woven textile from RPS‐20%−1.0 was wrapped with Kevlar fabric and subjected to a propane torch. The fabric protected a chocolate figurine for a burning time of 80 s (Figures 5b,c; Movie S4, Supporting Information) by limiting the upper surface temperature of the fabric to <60 °C, compared to 700–800 °C on the lower surface (Figure 5d). Importantly, both polyimide and silica aerogels are non‐flammable, and the composite fabric did not combust or disintegrate during and after the burn test. Post‐test analysis revealed that the Kevlar layer at the bottom burned through, but the RPS aerogel maintained its original shape (Figure S16a, Supporting Information) and showed low shrinkage even when treated with extremely high‐temperature conditions (Figure S17, Supporting Information), with carbonization of polyimide matrix transferring to compact structure, but silica aerogel particles retaining their porous structure (Figure S16b, Supporting Information). It is crucial to emphasize the composite aerogel's stability under wet conditions, particularly for applications such as firefighting suits. Benefiting from the inherent high hydrophobicity of both silica aerogel (mainly due to the surface modification with trimethylsilyl groups), and the moderate hydrophobicity of the rigid aromatic and methyl‐rich structure of the BTDA@TDI_MID polyimide,^[^ ^31^, ^32^ ^]^ the obtained composite aerogel demonstrates excellent hydrophobic properties. The RPS‐20%−1.0 aerogel fibers exhibit a contact angle of 118°, indicating strong hydrophobicity (Figure S18a, Supporting Information). Moreover, RPS‐20%−1.0 fabrics were subjected to heat treatments for 10 min at 150 and 300 °C, respectively, a standard condition according to our consultation with fire professionals,^[^ ^32^ ^]^ and then immersed in water: the fabrics still floated on the water surface after 12 h of immersion (Figure S18b, Supporting Information). The fabric maintains its stable hydrophobicity after high‐temperature treatment, with water absorption below 7% (Figure S18c, Supporting Information).

Li‐ion batteries can experience a thermal runaway to 800 °C,^[^ ^33^, ^34^ ^]^ causing deformation and stress within the battery pack, and a risk of propagation to adjacent cells. Aerogel thermal barriers can reduce the risk of thermal runaway propagation at a relatively low thickness that does not excessively reduce the volumetric energy density of the battery pack.^[^ ^35^, ^36^ ^]^ The flexible composite aerogel textile proves to be a suitable solution to address such challenges (Figure 5e). During thermal runaway in Li‐ion battery packs, massive gas production within the battery increases internal pressure, necessitating insulation materials that can maintain excellent thermal and mechanical properties under extreme conditions. As shown in Figure 5f and Figure S19 (Supporting Information), the surface temperature of the composite aerogel was over 500 °C lower than that of the hot plate under no pressure, indicating its outstanding high‐temperature insulation properties. Although the temperature difference across the composite aerogel decreases under high pressure, attributed to a combination of a decreased thickness and the partial degradation of its porous structure under the effect of high temperature and pressure, the composite aerogel still demonstrates excellent thermal insulation, maintaining a temperature difference of ≈250 °C between the upper and lower surfaces, highlighting its superior performance under high‐temperature and high‐pressure conditions. A woven aerogel fabric (RPS‐20%−1.0) with a thickness of 2.5 mm was positioned between two Li‐ion battery cells, and the surface temperatures and voltages of each battery were monitored (Figure 5g). A thermal runaway event was triggered or simulated by internal defects of the Li‐ion battery, heat accumulation during the rapid charging/discharging, or unpredictable mechanical, electrical, and thermal factors. The initial surface temperature of the runaway battery sharply increased to 800 °C within 80 s, while the Li‐ion battery under protection did not exhibit a temperature increase during this period. After heating for over 200 s, thermal failure occurred (Figure 5g). Correspondingly, the voltage of the burning battery dropped to zero when thermal failure occurred in ≈90 s, indicating complete battery failure. In contrast, the battery under protection only failed after 200 s of exposure (Figure 5h,i). While a sharp increase to 800 °C was identified within 2 s without any protection, and a quick complete battery failure was observed ≈100 s (Figure S20, Supporting Information). Thus, the composite aerogel fabric is an effective thermal management component to restrict thermal transfer between cells in Li‐ion battery packs. Particularly noteworthy is the fact that considering the working temperature of those specific applications (≈200 °C), owing to the high‐temperature resistance of the composites, the materials can still be fully recycled even after prolonged exposure to such temperatures exceeding 200 °C (Figures S14a–c, Supporting Information).

## 3 Conclusion and Outlook

In conclusion, utilizing Density Functional Theory (DFT) simulations, we have elucidated the mutual interaction forces of polyimides in various solvents. This study provides critical guidance for the design of recyclable polyimide composite aerogels, thereby enhancing their manufacturability for multiscale fabrication in 1D, 2D, and 3D geometries with excellent recyclability. Across all formulations, the engineered materials exhibit superior mechanical properties, exceptional thermal stability (both chemically and structurally), and outstanding thermal insulation properties. The design‐for‐disassembly approach of these composites enables complete recycling and separation of both polyimide and silica aerogel, achieving high yield without any degradation in material properties. The unique combination of lightweight design, high‐performance retention, and excellent recyclability positions this composite aerogel as a superior and sustainable choice for a wide range of high‐value applications. This is particularly relevant in emerging fields such as thermal runaway protection for Li‐ion batteries in electric vehicles and high‐performance textiles.

## Conflict of Interest

The authors declare no conflict of interest.

## Supporting information



Supporting Information

Supplemental Movie 1

Supplemental Movie 2

Supplemental Movie 3

Supplemental Movie 4

## Data Availability

The data that support the findings of this study are available from the corresponding author upon reasonable request.
