# Supplementary material for: Multiscale Manufacturing of Recyclable Polyimide Composite Aerogels
Source: Adv Mater. 2024 Nov 13;37(5):2411599. doi: 10.1002/adma.202411599 (PMC11795721; doi:10.1002/adma.202411599)
Supplement: Supplementary file 1 — Supporting Information [file ADMA-37-2411599-s001.docx]

Supporting Information

**Multiscale Manufacturing of Recyclable Polyimide Composite Aerogels**

*Mengmeng Li, Tingting Wu, Zhiyang Zhao, Lei Li, Tongxin Shan, Hui Wu, Robert Zboray, Francesco Bernasconi, Yongjie Cui, Peiying Hu, Wim J. Malfait,^*^ Qinghua Zhang,^*^ and Shanyu Zhao^*^*

M. Li, T. Wu, Z. Zhao, T. Shan, P. Hu, W. J. Malfait, S. Zhao

Laboratory for Building Energy Materials and Components, Swiss Federal Laboratories for Materials Science and Technology, Empa, Überlandstrasse 129, 8600 Dübendorf, Switzerland.

M. Li, Q. Zhang

College of Materials Science and Engineering, Donghua University, Shanghai 201620, PR China

T. Wu

Institute of Sustainability for Chemicals, Energy and Environment (ISCE^2^), Agency for Science, Technology and Research (A*STAR), 1 Pesek Road, Jurong Island 627833, Singapore

L. Li, H. Wu

State Key Laboratory of New Ceramics and Fine Processing, School of Materials Science and Engineering, Tsinghua University, 100084, Beijing, China

L. Li, T. Shan

National Engineering Research Center of Electric Vehicles, Beijing Institute of Technology, Beijing 100081, Beijing, China

R. Zboray

Center for X-ray Analytics, Swiss Federal Laboratories for Materials Science and Technology, Empa, 8600 Dübendorf, Switzerland

F. Bernasconi

Materials for Energy Conversion Laboratory, Swiss Federal Laboratories for Materials Science and Technology, Empa, 8600 Dübendorf, Switzerland; ETH Zürich, Department of Materials, 8093 Zürich, Switzerland

Y. Cui

School of Energy and Materials, Shanghai Polytechnic University, Shanghai 201209, China.

E-mail: shanyu.zhao@empa.ch, wim.malfait@empa.ch, qhzhang@dhu.edu.cn

**Materials and Methods**

Preparation of polyimide-silica aerogel dispersions

Firstly, soluble polyimide (copolyimide, *P84^®^*, Evonik-Degussa AG) solutions were prepared by adding the polyimide powder into the dimethylacetamide (DMAc, analytical reagent, purchased from Chemie-Brunschwig AG) and stirring. After it dissolved completely, the silica aerogel particles (amorphous, 5-20 µm, Cabot Aerogel IC3100) were added and stirred for 5 min. Then, the compounds were homogenized by a speed-mixing process (speed mixer, DAC 150.1 FVZ, FlackTek) with the program of 1000 rpm, 1500 rpm, 2000 rpm and 2350 rpm for 4 min, which was repeated twice. Finally, the achieved silica-polyimide mixture was further centrifuged (Rotina 380, Hettich) for 10 min at a speed of 3500 rpm to remove the bubbles. A series of dispersions were prepared by changing the contents of polyimide and silica aerogel, and all the samples were named using the following convention: RPS-x%-y denotes a dispersion with x wt% polyimide solution and y g silica aerogel powder, and the mass of the polyimide solution are all 20 g (Table S1).

Electrospinning of recycling polyimide-silica aerogel nanofiber membranes

A vacuum-defoamed RPS-20%-0.5 dispersion was placed in a 10 mL syringe and injected through a metal needle (21 G) at 3 µl/min by a syringe pump (MTI) mounted on the sliding arm (MTI). A potential of 10 kV was applied between the metallic needle and a rotating disk collector (MTI, 10 cm diameter) by two high voltage sources (GenVolt) at a distance of ~ 8 cm. The resulting fibers were collected for 2 hours on the collector rotating at 400 rpm to produce the composite membrane.

Wet-spinning of and weaving of recycling polyimide-silica aerogel composite aerogel fibers

A facile wet-spinning method combined with the supercritical CO_2_ drying technique was carried out for preparing RPS fibers. In detail, the spinning dispersion was extruded through an injection syringe with a nozzle diameter of 250 to 840 μm into an ethanol coagulation bath at a speed of 0.3~1 ml/min, and the as-spun fiber was collected. Subsequently, the gel fibers were immersed in fresh ethanol (with 5% isopropanol) for 24 h to remove residual DMAc. Afterward, the gel fibers were placed into a supercritical extractor (Separex), and exchanged into supercritical CO_2_ fluid over 6 h (120 bar, 80 °C), to obtain the RPS fibers. Next, a warp/weft knitting method was performed to obtain the RPS textiles: RPS fibers were set as the warp and other continuous aerogel fibers shut through the warp repeatedly, forming textiles.

Additive manufacturing of recycling polyimide-silica composite aerogel

A direct ink writing (DIW) method was performed for the printing. In detail, a cartridge with RPS-20%-1.9 dispersion was loaded in the printer (Bioplotter, Envision TEC) with a smooth flow-conical nozzle (410 µm, H. Sigrist & Partner). The ink was driven pneumatically through the nozzles with pressures ranging between 2.0 and 4.0 bar on a glass substrate (10 cm × 10 cm), with a filament extrusion rate of 15-24 mm/s. Afterwards, the printed polyimide-silica gels were placed in an ethanol coagulation bath with further ethanol washings to remove residual DMAc. Finally, supercritical CO_2_ drying was carried out in the same manner as for the fibers, to obtain the printed RPS composite aerogels with customized shapes.

Recycling the polyimide and silica aerogels

The good solubility of polyimide in selected solvents makes it possible to effectively separate the polyimide and silica aerogel by facile dissolution and filtration processes. Next, the obtained PI solution was subjected several times to ethanol as an anti-solvent to precipitate the PI, and then dried to recover the recycled polyimide. The collected silica aerogel was subjected to ethanol and supercritical CO_2_ drying to obtain recycled silica aerogel.

The recycling rate of polyimide and silica aerogel can be calculated using the following equation:

$$R\%=\frac{m_{R}}{m_{0}}\times100\%$$

R% is the recycling rate, m_R_ is the mass of the recycled polyimide (or silica aerogel) and m_0_ is the original mass of polyimide (or silica aerogel).

Rheology behavior

The rheometer (MCR 302 rheometer, Anton Paar) was used with a plate-plate (diameter of 25 mm) geometry with a gap of 0.5 mm. All the measurements were conducted at 20 °C. Different RPS dispersions were prepared for the rheological test in both rotation mode and amplitude sweeping mode. Storage and loss moduli were recorded with oscillatory strain variations from 0.01 to 1000% at a frequency of 1 Hz. The yield stress was defined as the shear stress where storage and loss moduli were equal. Viscosity was obtained by changing the rotational shear rate from 0.001 to 1000 s^-1^.

Microstructural analysis

The microstructure was observed on a field-emission SEM (FEI Nova NanoSEM 230) at an accelerating voltage of 10 kV and a working distance of ~5 mm. A Pt layer of ~15 nm (flat surface equivalent) was coated to guarantee a good electrical conductivity of the samples. Nitrogen sorption analysis was carried out on a nitrogen sorption analyzer (TriFlex, Micromeritics) after prior degassing for 900 min at 80 °C and 0.02 mbar. The specific surface areas and pore volume were derived by the BET method and the pore size distributions were calculated by the BJH method.

Density and porosity

The apparent density (*ρ_a_*), skeleton density (*ρ_s_*) and porosity were calculated by following formulas:

$\text{ρ}\text{a}\text{=}\frac{\text{m}\text{T}}{\text{πr}\text{2}\text{×L}}$ (1)

$\text{ρ}\text{s}\text{=}\frac{\text{m}\text{P}\text{×}\text{ρ}\text{P}}{\text{m}\text{T}}\text{+}\frac{\text{m}\text{S}\text{×}\text{ρ}\text{S}}{\text{m}\text{T}}$ (2)

$\text{Porosity(\%)=}\left( \text{1-}\frac{\text{ρ}\text{a}}{\text{ρ}\text{s}} \right)\text{×100\%}$ (3)

In the formulas, m_T_ is the total weight of the sample; m_P_ and m_S_ are the weights of polyimide and silica aerogel, respectively. r is the radius of the fiber and L is the length of the sample. ρ_RP_ (1.42 g/cm^3^) and ρ_S_ (1.69 g/cm^3^) are the skeleton densities of polyimide and silica aerogel which were determined with a helium pycnometer (AccuPyc II 1340, Micromeritics, USA) equipped with a 1 cm^3^ sample chamber.

Mechanical properties

The tensile strength of the composite aerogel fibers was operated on a universal testing machine (Zwick 1484, Zwick GmbH, Germany) at a tensile rate of 10 mm/min, and every sample was tested five times. Identical cylinders (8 mm ×5 mm) were printed with a 410 μm nozzle for compression tests, which were carried on the same equipment as the tensile strength test with a compression rate of 1.0 mm/min up to 70% compressive strain.

Hydrophobicity

The hydrophobicity of RPS-20%-1.0 fiber was evaluated through water contact angle measurements using a Contact Angle System OCA (Dataphysics TBU 90E, Germany), with a high-speed camera and a precision stainless steel tip (Gauge 32, EFD). Water was selected as the solvent and the volume of the water droplet was 10 μL. RPS-20%-1.0 fabrics were heat-treated at 100°C, 200°C, and 300°C for 10 minutes in an air atmosphere, cooled to room temperature, and subsequently immersed in deionized water for 12 hours. Next, the water on the fabric surfaces was gently wiped off using filter paper.

$$\text{Water sorption(\%)=}\left( \frac{\text{m-}\text{m}_{0}}{\text{m}\text{0}} \right)\text{×100\%}$$

In the formulas, m represents the weight of the RPS-20%-1.0 fabrics after the water sorption process, while m₀ denotes the weight of the fabric before the water sorption processes.

Thermal conductivity

Identical square planar (width: 55 mm; thickness: 10 mm) from RPS-15%-1.9 was cast for the thermal conductivity test. After gelation, solvent exchange and supercritical CO_2_ drying, the aerogel monolith was evaluated by a custom-built guarded hot plate device (guarded zone: 50 × 50 mm^2^, measuring zone: 25 × 25 mm^2^) for the thermal conductivity.

Thermal stability analysis

The thermal stability of composite aerogels was evaluated by a thermogravimetric analyzer (Netzsch TG 209F1) at 10 ^o^C/min in a reconstituted air atmosphere.

Chemical structure

ATR-FTIR was performed on a spectrometer (Bruker Switzerland AG with the ATR name Tensor 27) with the wavenumber range of 500~4000 cm^-1^ to confirm the chemical structure of the original and recycled silica aerogel, polyimide and their composites.

^1^H and ^13^C NMR spectra were acquired with a Bruker Advance III system with a wide-bore 9.4 T magnet corresponding to Larmor frequencies of 400.2 MHz for ^1^H and 100.6 MHz for ^13^C, equipped with a 5 mm CryoProbe™ Prodigy probe equipped with z-gradient. The original and recycled PI were dissolved in fully deuterated DMF at a concentration of ~60 mg of PI per ml of solvent. The measurements were performed at 298 K using standard Bruker pulse programs and parameter sets applying 8 (30)° pulse lengths of 1.0 (3.3) µs and relaxation delays of 4 (2) s, and 16 (1024) scans were accumulated for ^1^H (^13^C) NMR data.

Molecular weight and distribution analysis

Gel permeation chromatography (GPC, Agilent PL-GPC50) was carried out with a mobile phase of dimethyl sulfoxide (DMSO) and a standard sample of poly(methyl methacrylate) (PMMA) to character the molecular weight of the original and recycled polyimide. The contraction of the polyimide is 2.5 mg/mL, the flow rate is 1.0 mL/min.

Density functional theory (DFT) simulation

All the calculations of the model compounds studied in this work were performed using the Gaussian 16 software package^[S1]^. The alkyl side chains are replaced with methyl groups to save computation time without affecting the description of electronic properties. The optimal conformations for polyimides (BTDA@TDI_MDI, Kapton and Upliex), DMAc, NMP and H_2_O were calculated by DFT at the B3LYP/6-31G (d, p) level. In addition, the composite structures were calculated by DFT at the B3LYP-D3/6-31G (d, p) level. Then the ESP characteristic parameters and the RDG analysis were calculated based on the optimal structure with electronic wave function information using the Multiwfn 3.7(dev) program^[S2]^. The molecular surface electrostatic potential distribution is projected onto a Van Der Waals surface (electron density equivalent surface of 0.001 a.u.) by Gauss View 6.0 simulations. The nuclei and electrons of the molecule generate an electrostatic potential (*V*(r)) at each point r in the surrounding space, strictly given by the following equation:

$V\left( r \right)= \sum_{A} \frac{Z_{A}}{R_{A}-r}- \int\frac{\rho(r^{'})dr^{'}}{\left| r^{'}-r \right|}$ (3)

where *Z*_A_ and *R*_A_ denote the nuclear charge and position vector of atom A respectively. *ρ*(r) is the electron density of the molecule. ESPmin and ESPmax are the minimum and maximum values of the molecular surface electrostatic potential (fig. S1B).

Molecular polarity index (MPI) is calculated quantitatively based on the distribution characteristics of the electrostatic potential on the surface of a molecule. The value of MPI can be obtained by the following equation:

$\mathrm{MPI}=(1/A)\iint_{S} |V\left( r \right)|dS$ (4)

where *V*(r) is the surface electrostatic potential of the molecule and A is the surface area of the molecule. The greater the Molecular Polarity Index (MPI), the greater the overall polarity of the molecule.

X-ray tomographic microscopy

The 1D fiber (RPS-20%-1.0), 2D nanofiber membrane (RPS-20%-0.5) and 3D printed object (RPS-15%-1.9) were cut and tested by micro/nano computed tomography. A laboratory micro/nano CT scanner (EasyTom XL, RX Solutions, Chavanod, France) was utilized to acquire images of each sample. The scanner features a transmission nano-focus tube operated at 95 kVp and 30 µA tube current. It is combined with a high-resolution CCD camera coupled to a 20-µm thick Gadox scintillator through optical fibers, having a detection area of 36 mm × 24 mm with a pixel size of 18 µm. Scans were performed by taking 2016 projections over 360 degrees, with 5 frames of 1.5 s exposure time averaged for each projection. The voxel size of the scans varied 0.4^3^ μm^3^ -0.9^3^ μm^3^. Before reconstruction using the FDK algorithm^[S3]^, the projection images underwent phase retrieval using the Paganin phase-retrieval method^[S4]^. The phase retrieval parameters were lumped and expressed in terms of -6 dB point of the Paganin filter which was set to 0.2 of the Nyquist frequency. This propagation-based phase contrast enables to improve the contrast-to-noise ratio of the images significantly. The nanc-CT Images have been processed by the commercial package VG Studio Max 3.5 (Volume Graphics, Heidelberg, Germany)

Thermal protection test under high temperature and high pressure

The samples of RPS-20%-1.0 with dimensions of 50 mm × 50 mm × 7 mm were tested on a thermal runaway test setup (Hydraulic bench for the compression test device, EP-10XL, Transcell, America; Digital thermostat, 287 PID, Shenzhen Fan and Air Electronic Technology Co. Ltd, China). Real-time temperatures on both the hot and cold sides were recorded using a thermocouple thermometer (TASI, TA612C, China). Referring to standards GB228-87, GB228-2010, and GB7314-87, a standard procedure was applied to test the thermal insulation performance of the composite aerogel under high-temperature (650 ^o^C) and high-pressure conditions:

(1) Set the target force to 5760 N;

(2) Maintain the displacement for 180 s;

(3) Release the load to 200 N and hold for 2000 s;

(4) Return to the starting position.

In a second round of measurements, the effect of temperature on the insulation performance was tested on thinner samples (2.5 mm) using the same device (but without pressure) at 150°C and 500°C for 30 minutes, respectively.

Li-ion battery package thermal runaway protection test.

Li-ion battery package thermal runway protection test was evaluated by a homemade equipment. Two Li-ion cells (commercial Li-ion polymer cells with ternary materials cathode and graphite anode, 4400 mAh, 3.0-4.2 V) were placed on a heat plate with a temperature control box, and an RPS aerogel plate was put between the two cells. Four K-type thermocouples were fixed on each surface of the Li-ion cells to detect the real-time temperature. A data logger was used to record the voltage and surface temperature of the cells, and the sampling interval was set to 1 s. The whole experimental process consists of the following steps: (1) Cell conditioning: Before the overheat tests, the cells were charged using constant current (0.5C, 2.2 A) and constant voltage (4.2 V) mode (CC-CV mode) and discharged using constant current (0.5C, 2.2 A) mode (CC mode), and the cells were rested for 60 min between each step. Then, all the cells were charged to 100% SOC with CC-CV mode and rested for at least 90 min. (2) Overheat-to-thermal runaway: Before testing, thermocouples were fixed on the surface of the Li-ion cells, and then the cells were put on the heating plate and made sure that the broad surface of the cell was in contact with the heating plate. During the test, the temperature of the heating plate was controlled by a temperature control box; the heating power was set to 1140 W, which was the default control mode for the controller. The heating process was stopped when the voltage of the abused cell dropped to 0, which meant the failure of the cell. (3) After thermal runaway occurred, these cells were allowed to cool naturally at ambient temperature (about 25 °C) for disassembly. It should be noted that special care must be taken to ensure the safety of the operators and the equipment due to the potential risks of battery thermal runaway.

Thermal management application demonstration

Composite aerogel textiles based on RPS-20%-1.0 fibers with a thickness of 9 mm covered by a Kevlar fabric (0.25 mm) were burned or heated by a propanol torch with a distance of 15~25 cm, keeping the outer flame touching the fabric. A fox-shaped chocolate was placed on top of the composite textiles to test their thermal insulation properties. The results were monitored with thermocouples (k type directly to the flame, and t type above the composite) and infrared imaging, and a chocolate fox was also placed on the top side to be protected by the composite. During the test, infrared images of the composite fabric surface were recorded by an infrared thermal imager (Testo 880-1). The 3D printed RPS-15%-1.9 honeycomb was burned by the outer flame of propanol torch for 2 min.


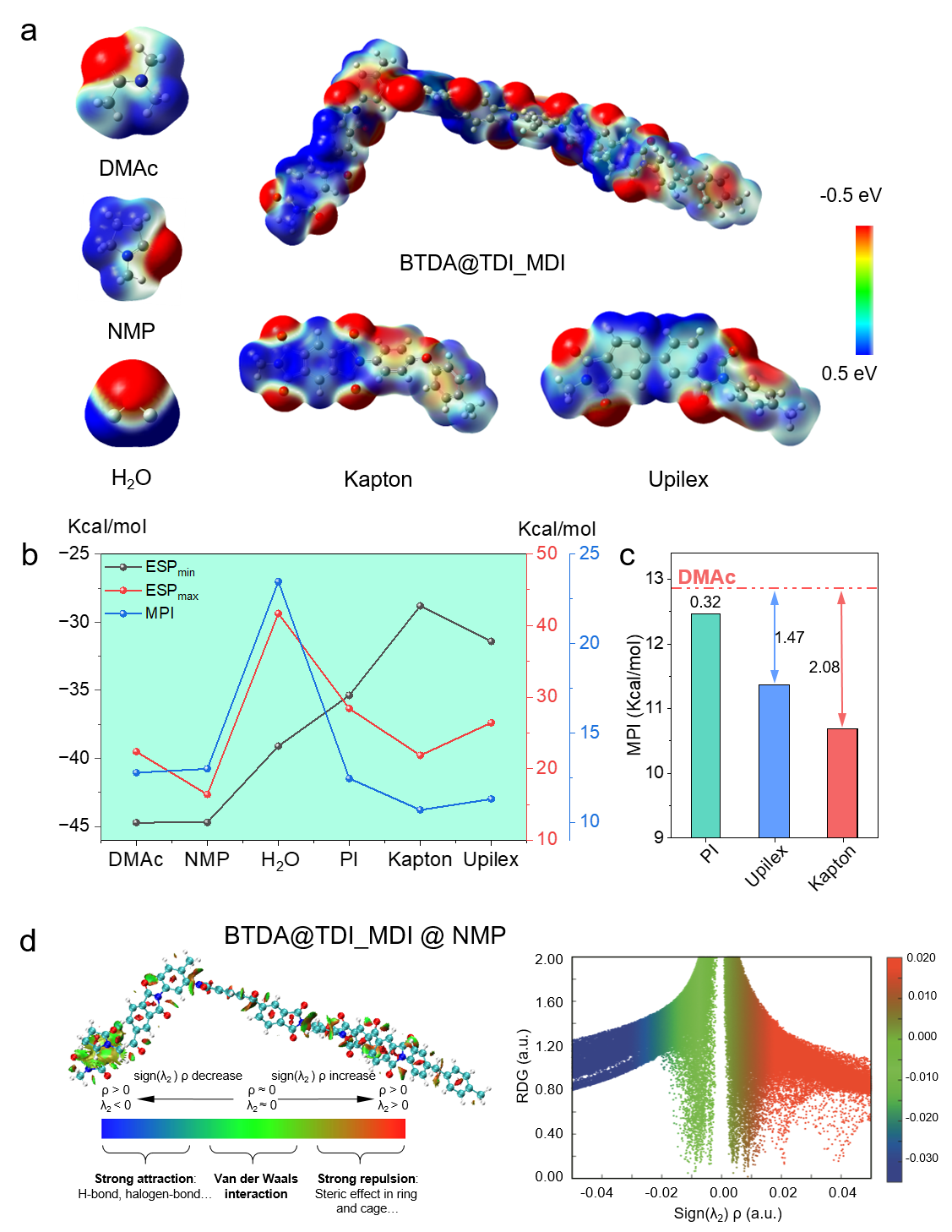


**Figure S1**. (a) Electrostatic potential distributions of different solvents and polyimides. (b) ESPmin, ESPmax and MPI of different solvents and polyimides based on the molecular simulation. (c) Difference of MPI between polyimides and DMAc. (d) Schematic diagram of the weak interaction between polyimide and NMP and the integration result.


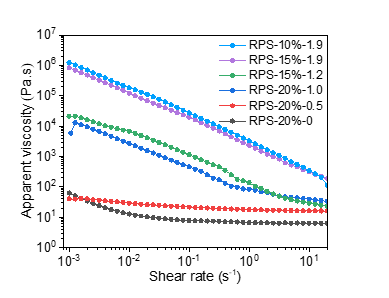


**Figure S2**. Steady-shear rheology of polyimide-silica dispersions with different filler contents.


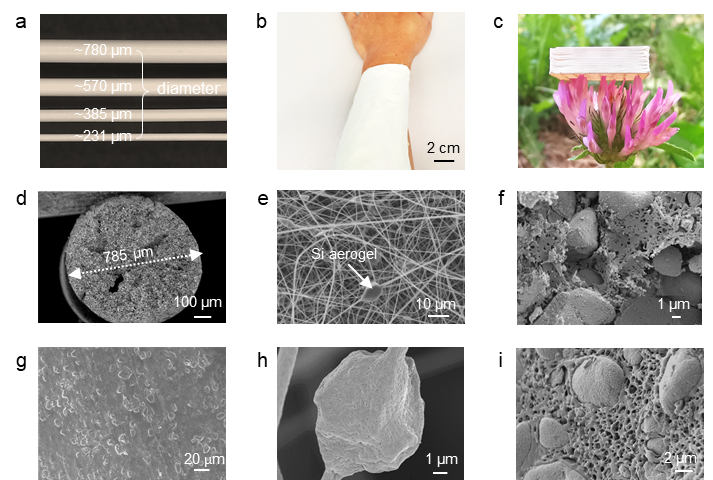


**Figure S3**. Pictures of (a) RPS aerogel fibers with different diameters, (b) the RPS nanofiber film and (c) printed RPS aerogel. Cross-section SEM images of (d) RPS-20%-1.0 aerogel fiber (e) nanofiber in the RPS-20%-0.5 nanofiber membrane and (f) the printed RPS-15%-1.9 object. Surficial SEM images of (g) RPS-20%-1.0 aerogel fiber, (h) nanofiber in the RPS-20%-0.5 nanofiber membrane and (i) the printed RPS-15%-1.9 object.


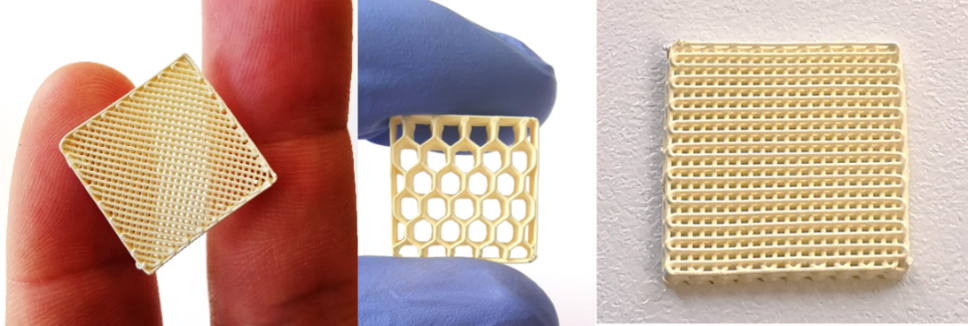


**Figure S4**. Printed RPS composite aerogels with different patterns (20 mm × 20 mm).


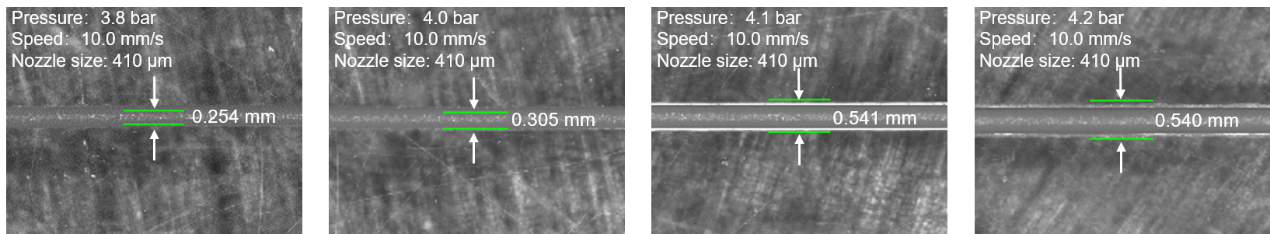


**Figure S5**. RPS-15%-1.9 inks printed at different pressures with a nozzle size of 410 μm and printing speed of 10.0 mm/s.


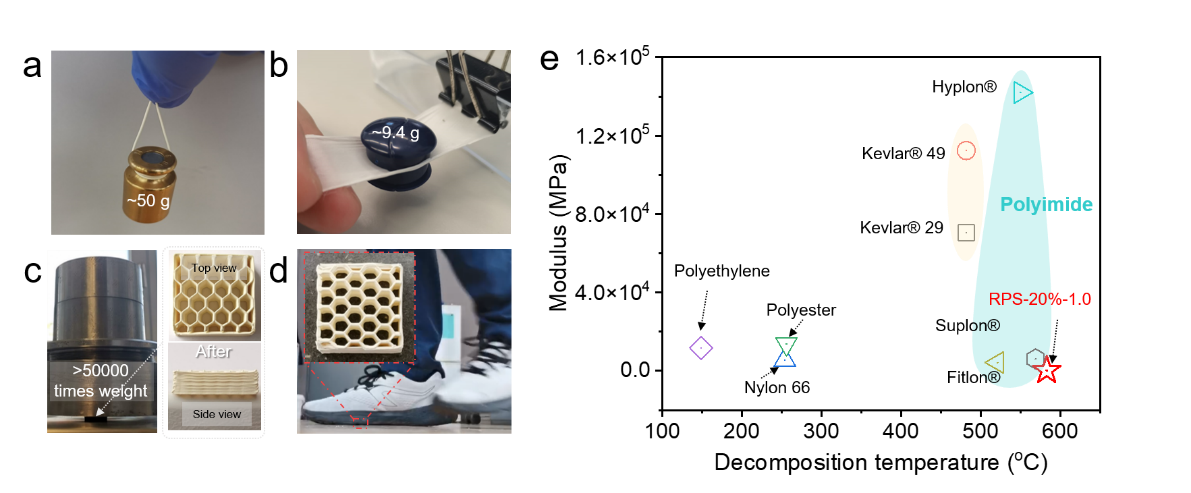


**Figure S6**. (a) Picture of RPS-20%-1.0 fiber holding a 50 g weight. (b) Picture of electrospun RPS-20%-0.5 nanofiber membrane withstanding a 9.4 g magnet. (c) The printed RPS-15%-1.9 aerogel honeycomb bears more than 50,000 times its weight and (d) even an adult (~80 kg). (e) Comparison of RPS-20%-1.0 with the commercial fibers in terms of mechanical properties and decomposition temperature.


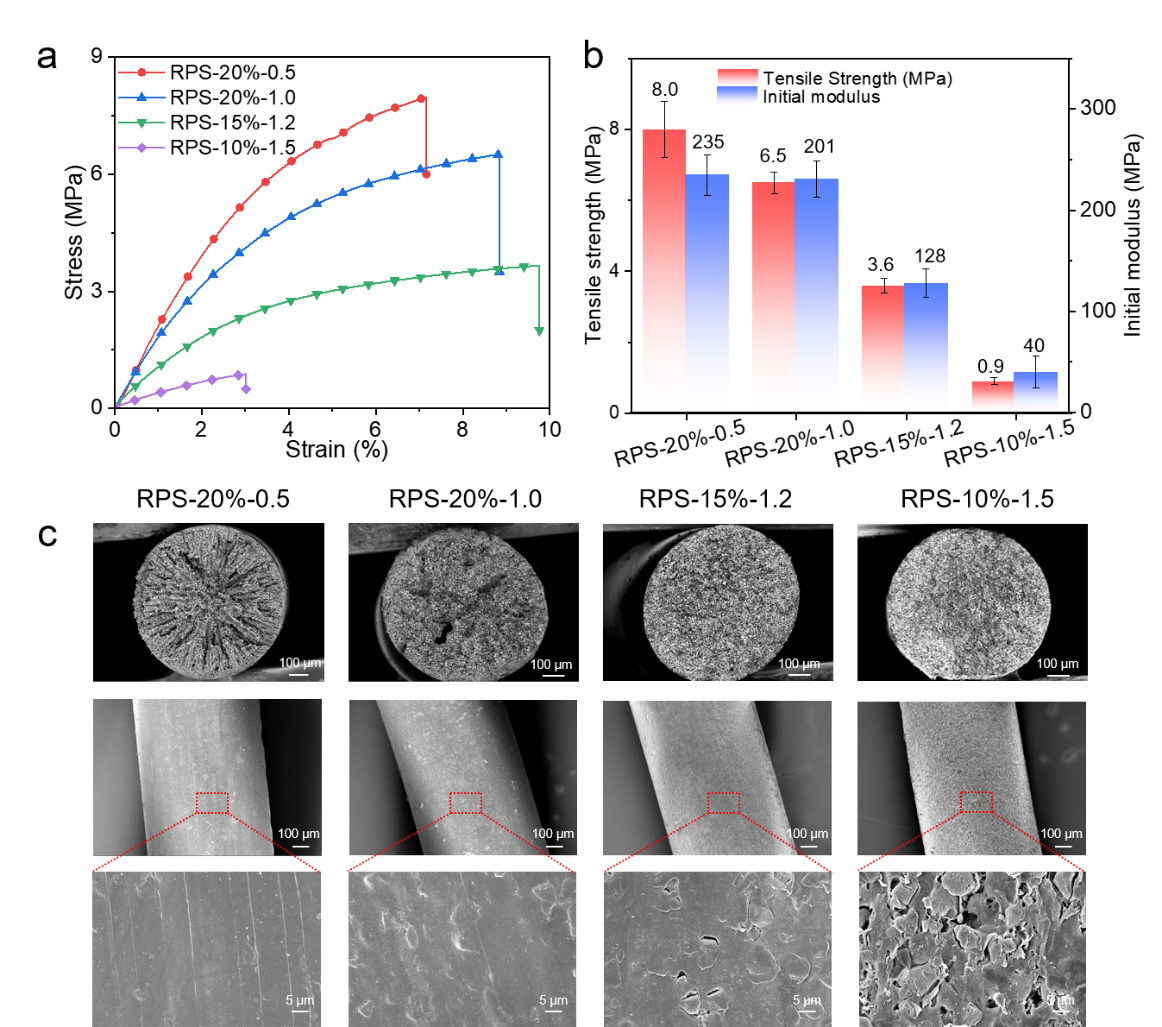


**Figure S7**. (a) Stress-strain curves of RPS aerogel fibers. (b) Mechanical properties of RPS aerogel fibers. (c) Cross-sectional and surficial SEM images of RPS aerogel fibers.


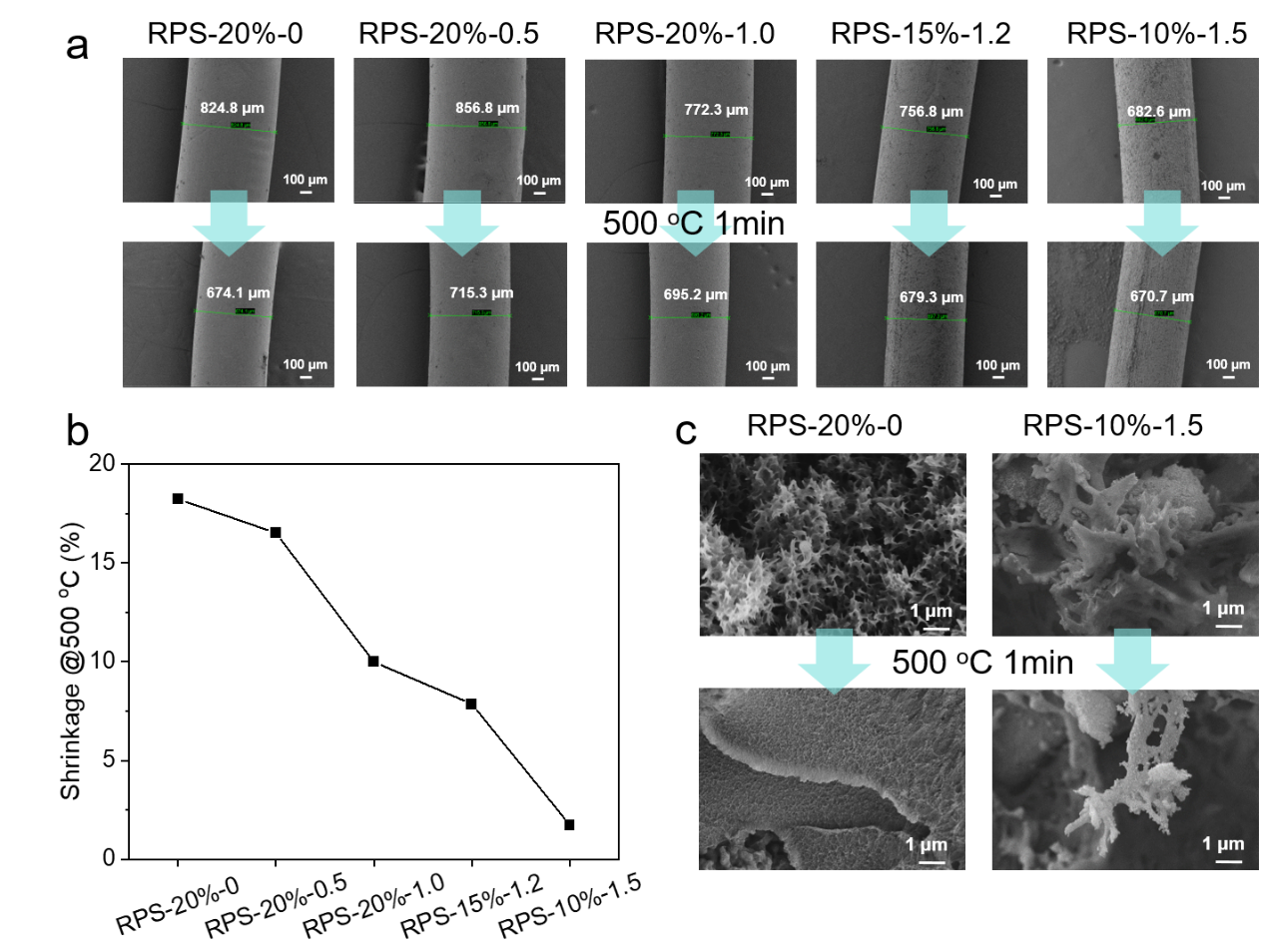


**Figure S8**. (a) SEM images of different RPS aerogel fibers before and after the treatment at 500 ^o^C for 1 minute. (b) Linear shrinkage (diameter) of different RPS aerogel fibers before and after the treatment at 500 ^o^C for 1 minute. (c) Cross-section SEM images of RPS-20%-0 and RPS-10%-1.5 before and after the treatment at 500 ^o^C for 1 minute.


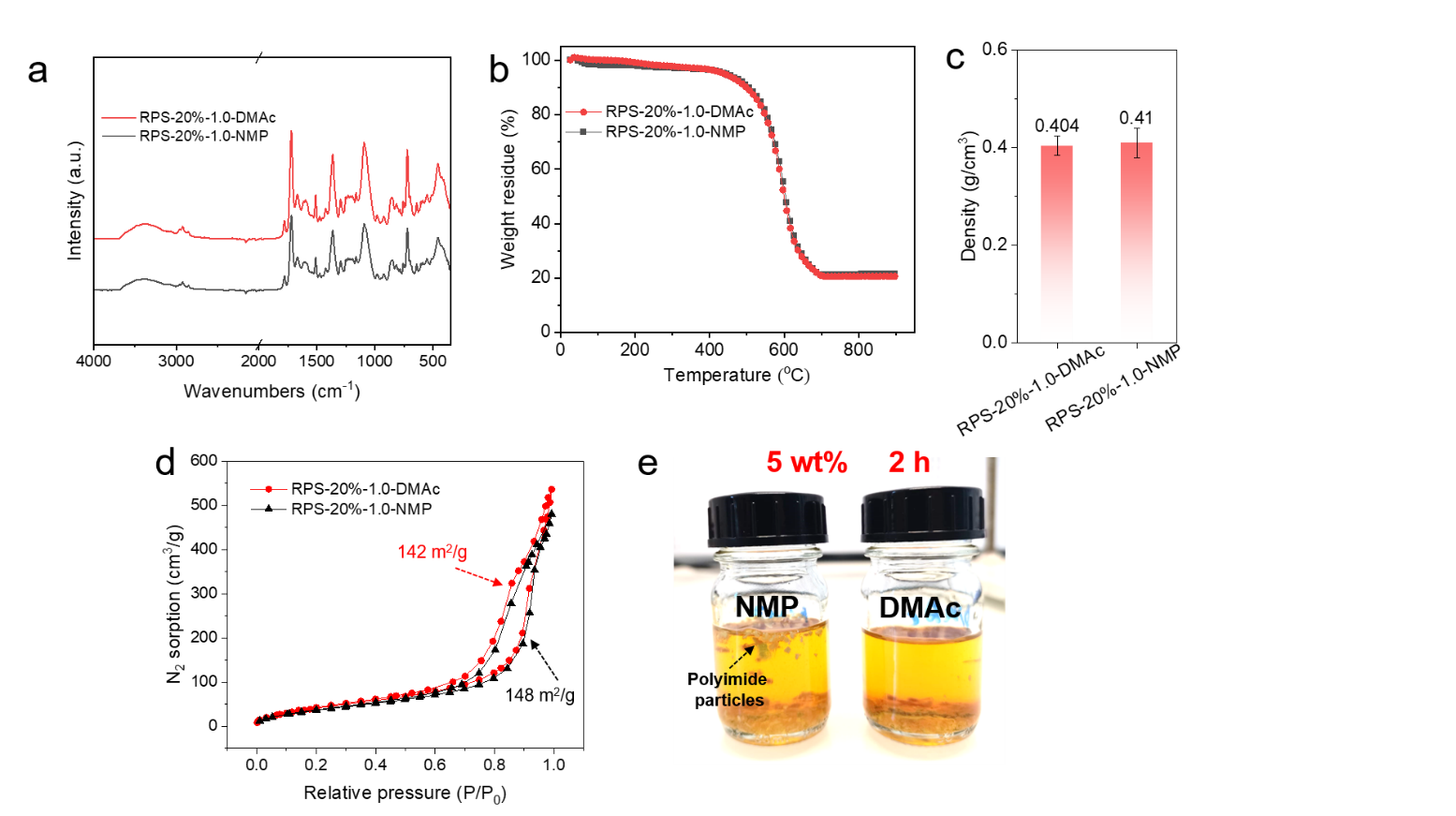


**Figure S9**. (a) FTIR spectra of RPS-20%-1.0 prepared from NMP and DMAc. (b) TGA curves of RPS-20%-1.0 prepared from NMP and DMAc. (c) Density of RPS-20%-1.0 from NMP and DMAc. (d) N_2_ sorption isotherms RPS-20%-1.0 prepared from NMP and DMAc. (e) A photograph of 1 g of polyimide placed in 20 ml of NMP and DMAc for 2 h, respectively.


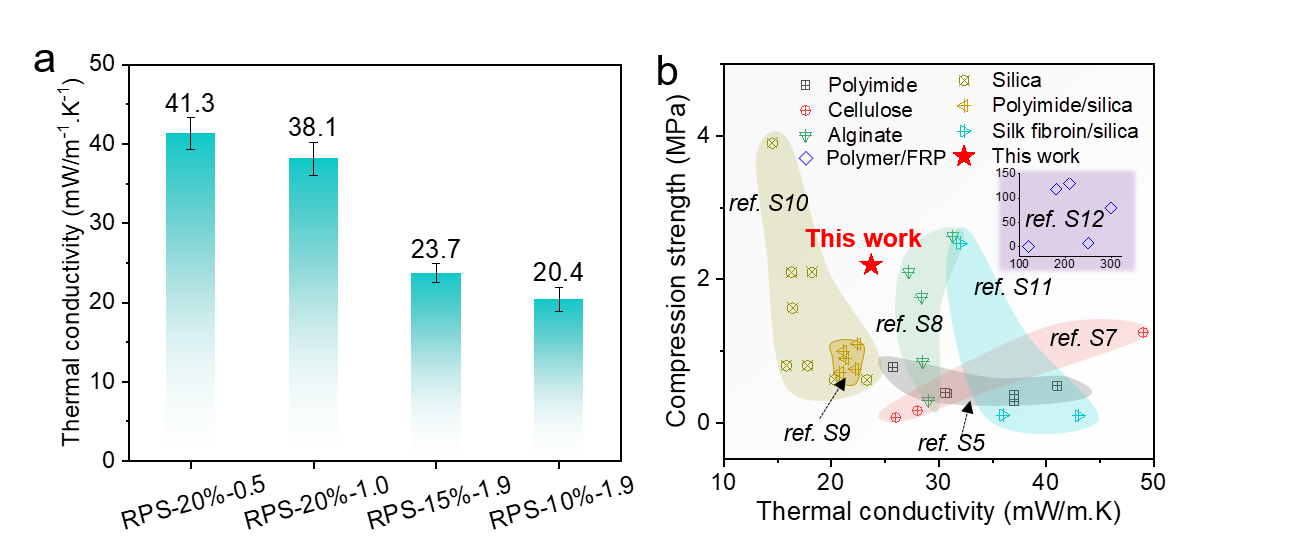


**Figure S10**. (a) Thermal conductivity of composite aerogels. (b) Comparison of the aerogels in terms of density and compressional Young's modulus.


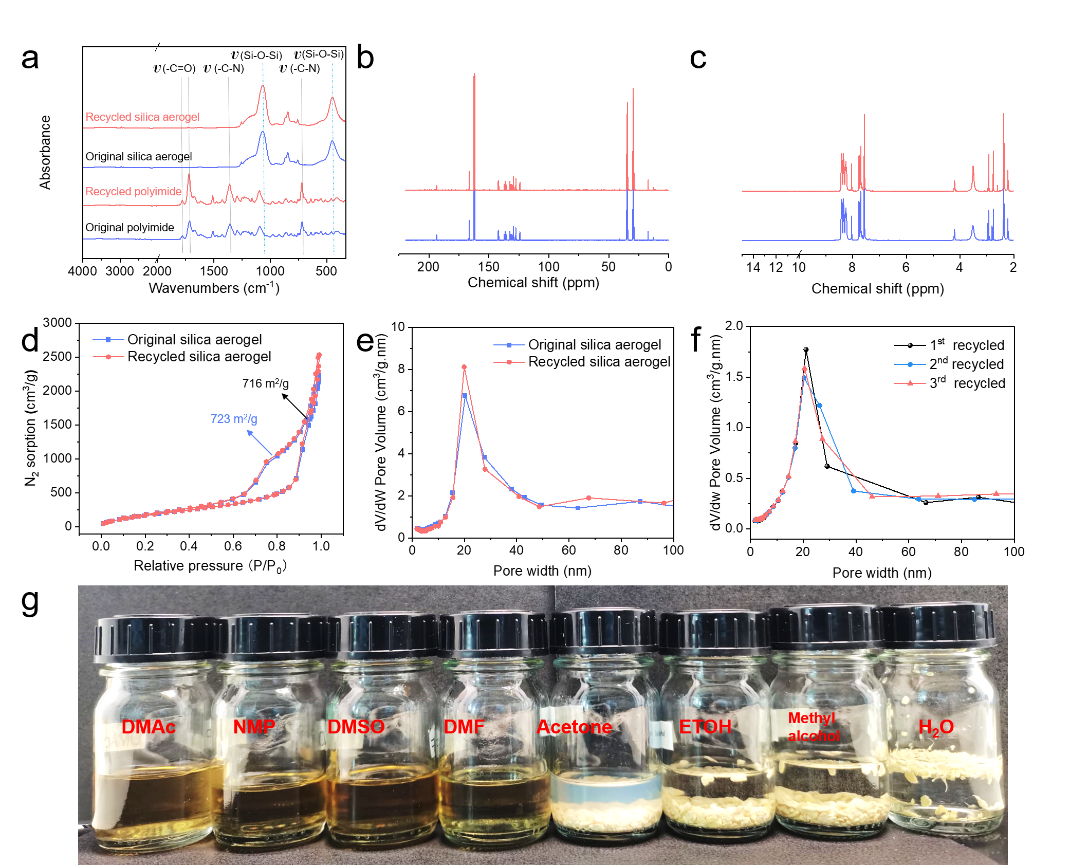


**Figure S11**. (a) FTIR spectra of the original and recycled polyimide and silica aerogel. (b) ^13^C and (c) ^1^H NMR of the original and recycled polyimide. (d) N_2_ sorption isotherms and (e) pore size distribution of the recycled silica aerogel and pristine silica aerogel by BJH method. (f) Pore size distribution of N_2_ sorption isotherms of the recycled RPS composite aerogels and sample treated at 200 ^o^C in an air atmosphere. (g) Picture of BTDA@TDI_MDI polyimide in different solvents.


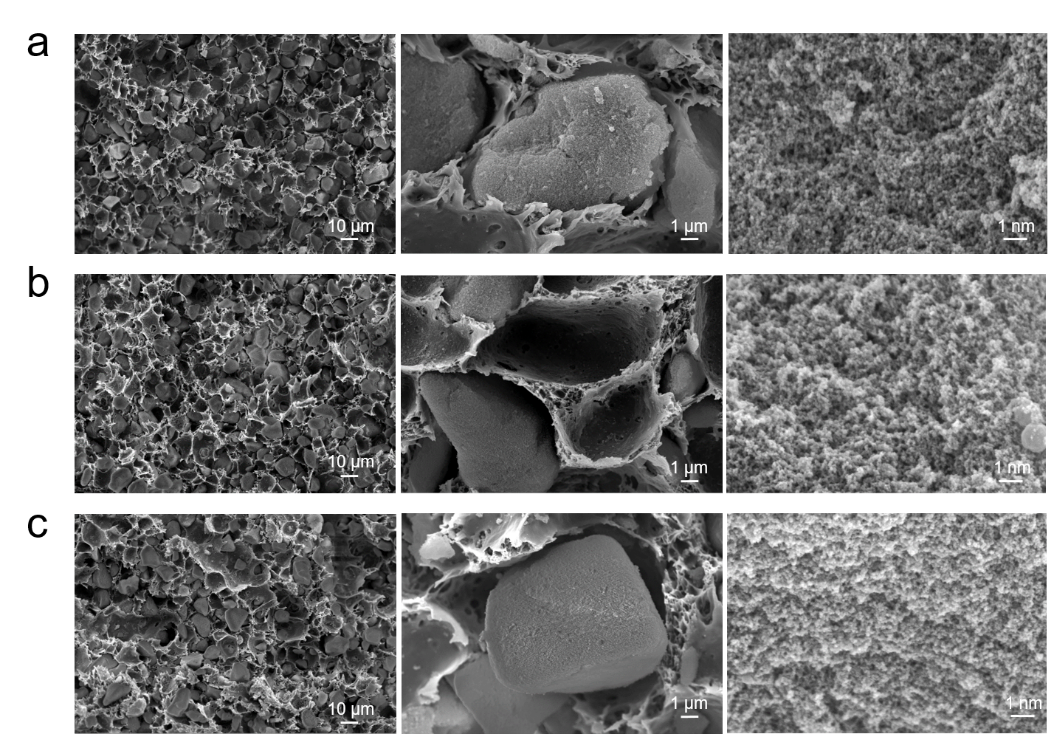


**Figure S12**. Cross-section SEM images of (a) 1^st^ recycled (b) 2^nd^ recycled and (c) 3^rd^ recycled RPS aerogel fiber with different magnifications.


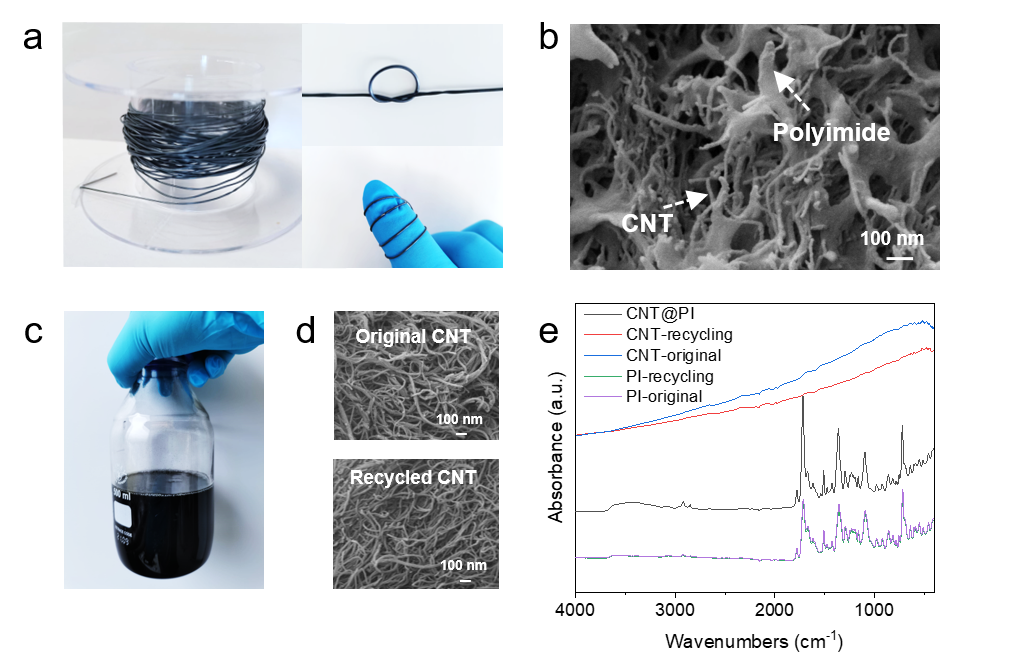


**Figure S13**. (a) Pictures of CNT@polyimide composite aerogel fibers. (b) Cross-section SEM image of CNT@polyimide composite aerogel fiber. (c) Dispersion of polyimide and CNT in DMAc during the recycling process. (c) SEM images of the original and recycled CNT. (d) FTIR spectra of polyimide, CNT and the prepared CNT@polyimide composite aerogel fiber.


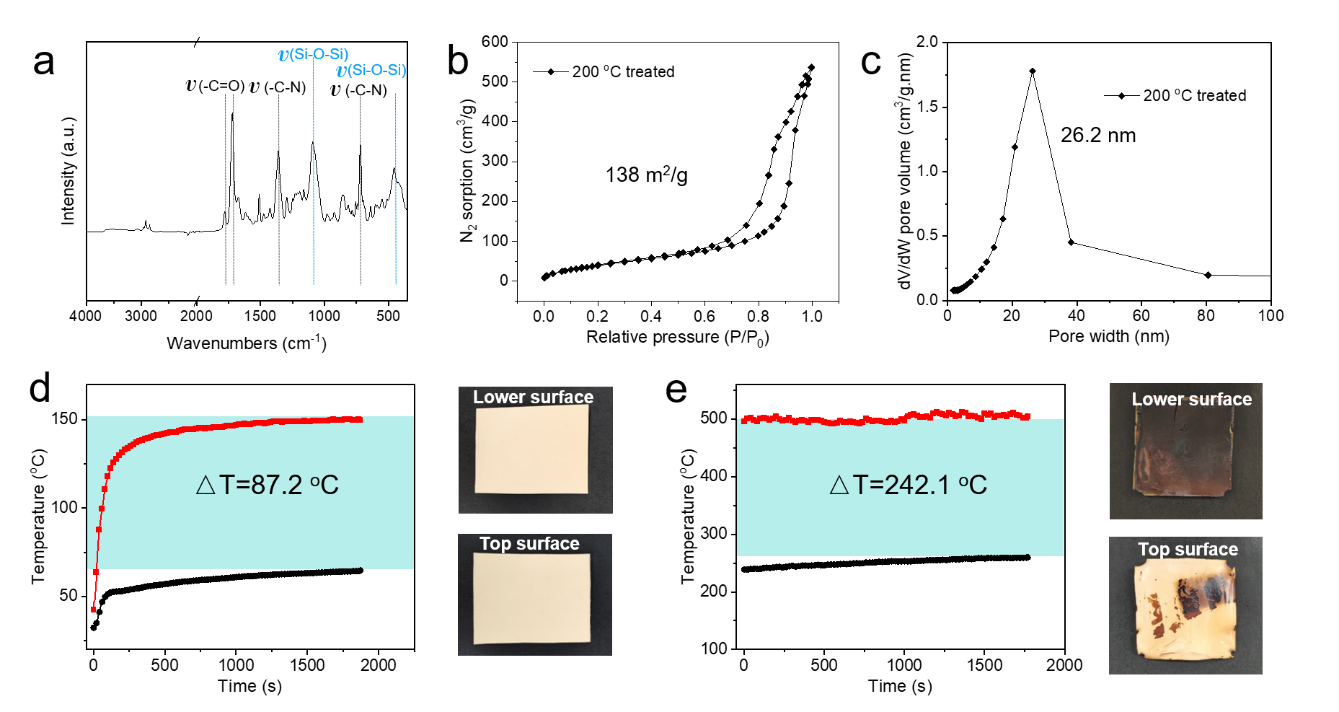


**Figure S14**. (a) FTIR spectra, (b) N_2_ sorption isotherms and (c) pore size distribution of the RPS aerogel treated at 200 °C in an air atmosphere. (d) Real-time temperature curves of a cast RPS-20%-1.0 aerogel (black=top surface, red=lower surface) when the lower plate is heated to 150 ^o^C and their surface pictures after the test. (e) Real-time temperature curves of the RPS-20%-1.0 aerogel (black=top surface, red=lower surface) when the lower plate is heated to 500 ^o^C and their surface pictures after the test.

After ~30 minutes at 150°C, the surface temperature difference reached ∆T=87°C, with no visible shrinkage or decomposition. The composite aerogel maintained excellent thermal insulation even at 500°C for 30 minutes, with ∆T=242°C. The black color of the hot side surface was observed due to surface carbonization of polyimide under long heat exposure, the cold side surface remained unchanged, demonstrating the composite aerogel’s strong resistance to high temperature.


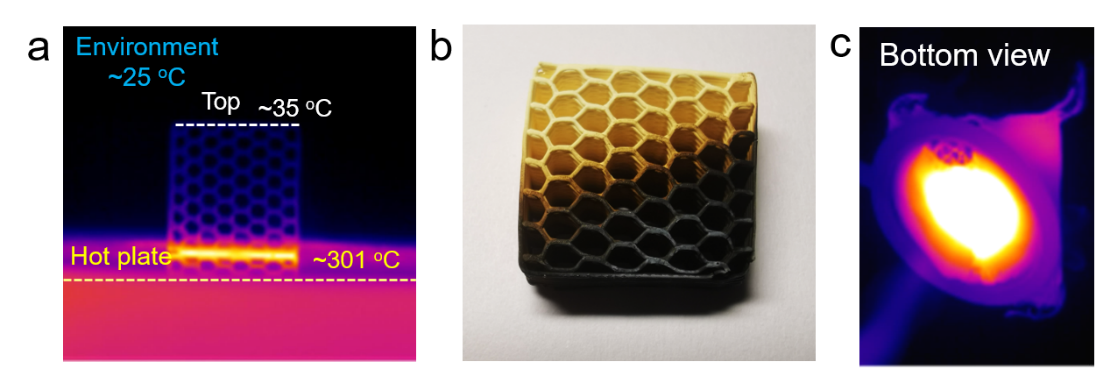


**Figure S15**. (a) Infrared image of the 3D printed honeycomb under 300 °C. (b) Picture of the 3D printed honeycomb after being burned by a blast burner for 30 s. (c) Infrared images of the composite aerogel fabric during the fire test.


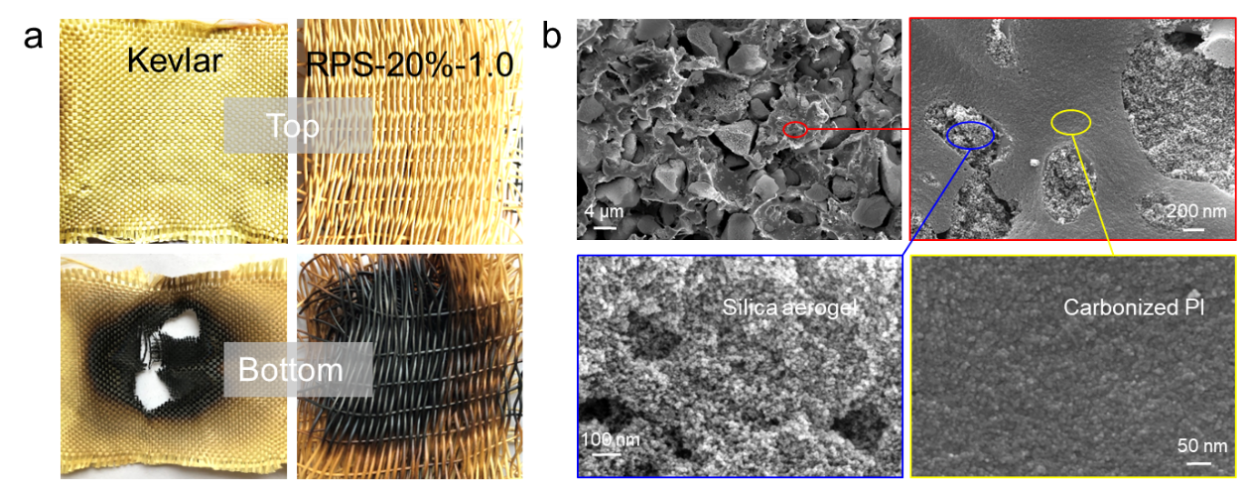


**Figure S16**. (a) Picture of the Kevlar and RPS-20%-1.0 fabric from the top and bottom surface after the fireproof test. (b) Cross-section SEM images of RPS-20%-1.0 fiber with different magnifications after the burnt test.


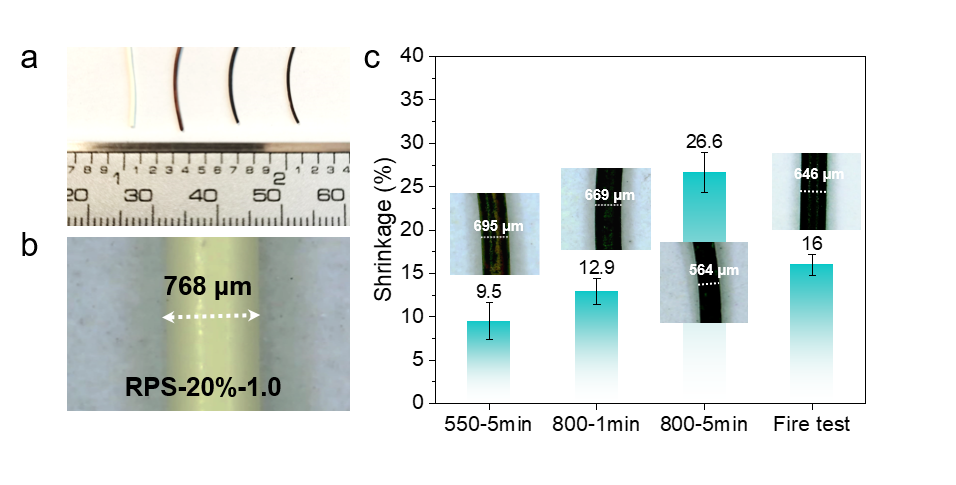


**Figure S17**. (a) Picture of RPS-20%-1.0 treated with different temperatures. (b) Microphotograph of the original RPS-20%-1.0 aerogel fibers. (c) Shrinkage of RPS-20%-1.0 treated with different temperatures.


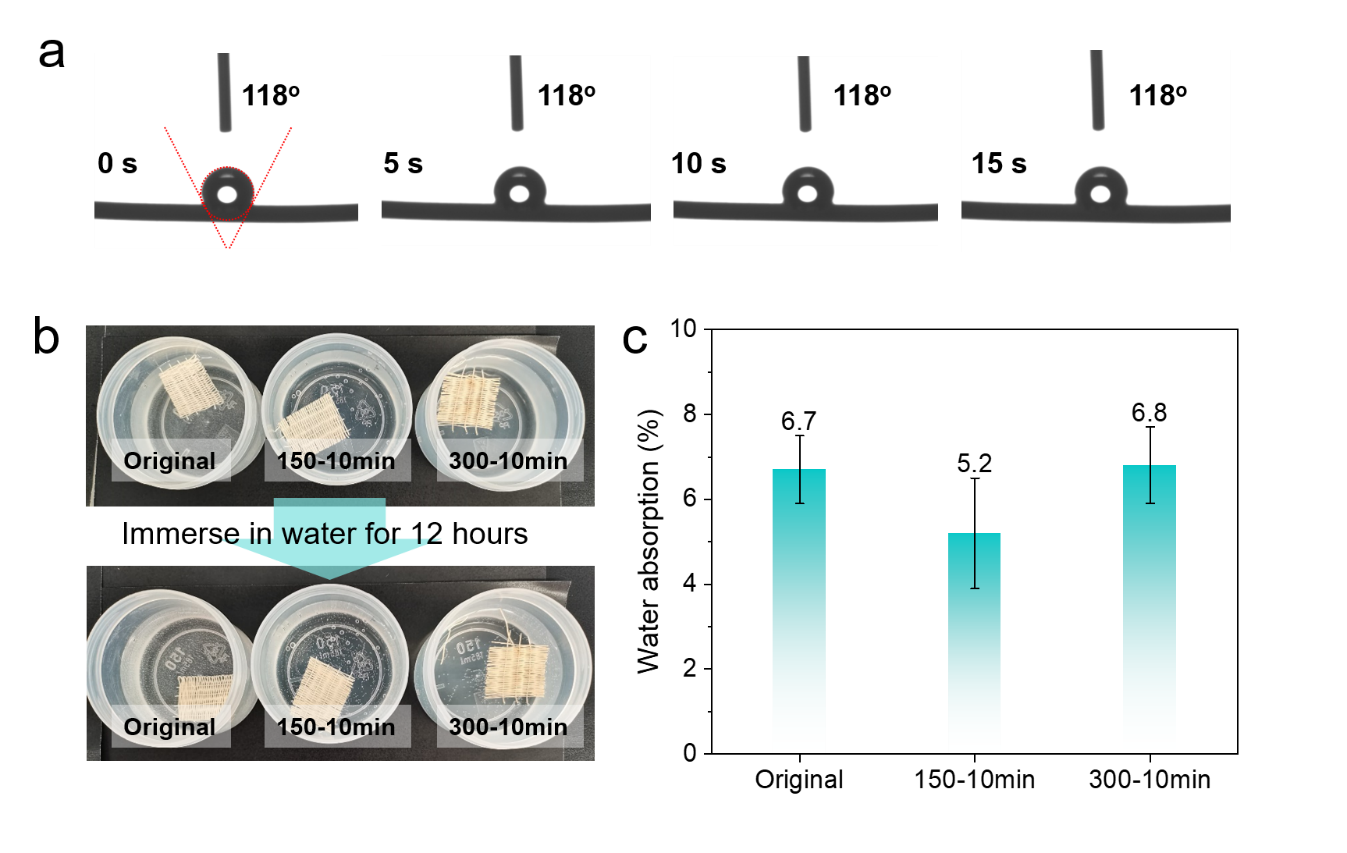


**Figure S18**. (a) Water contact angles of RPS-20%-1.0. (b) Pictures of the water absorption test of the RPS-20%-1.0 fabrics treated under different temperatures (150 and 300°C). (c) Water absorption of RPS-20%-1.0 fabrics treated under different temperatures after immersion in water for 12 h.


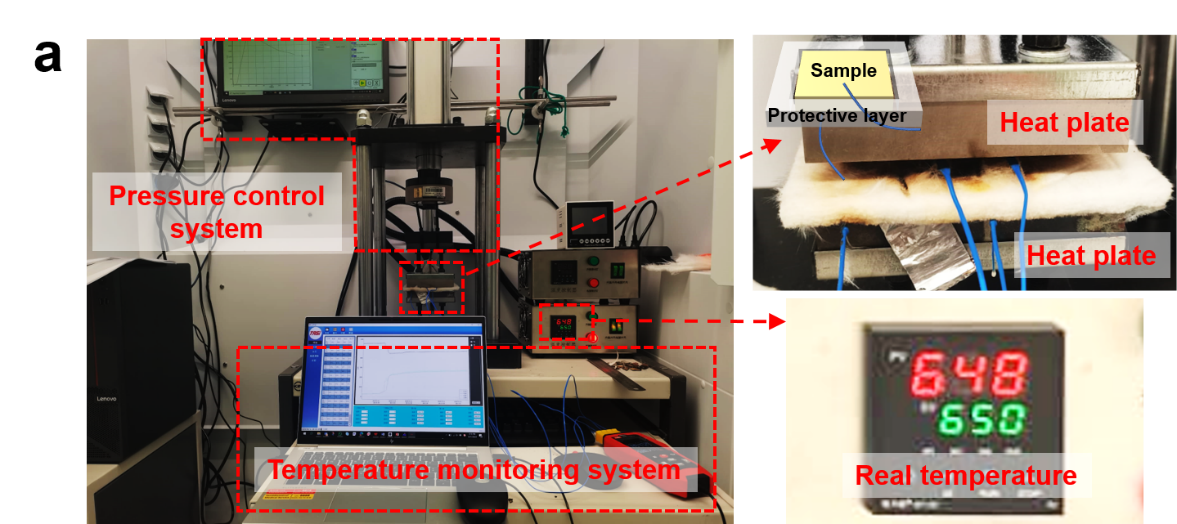


**Figure S19**. Schematic diagram of testing the thermal insulation performance of the composite aerogel under pressure conditions.


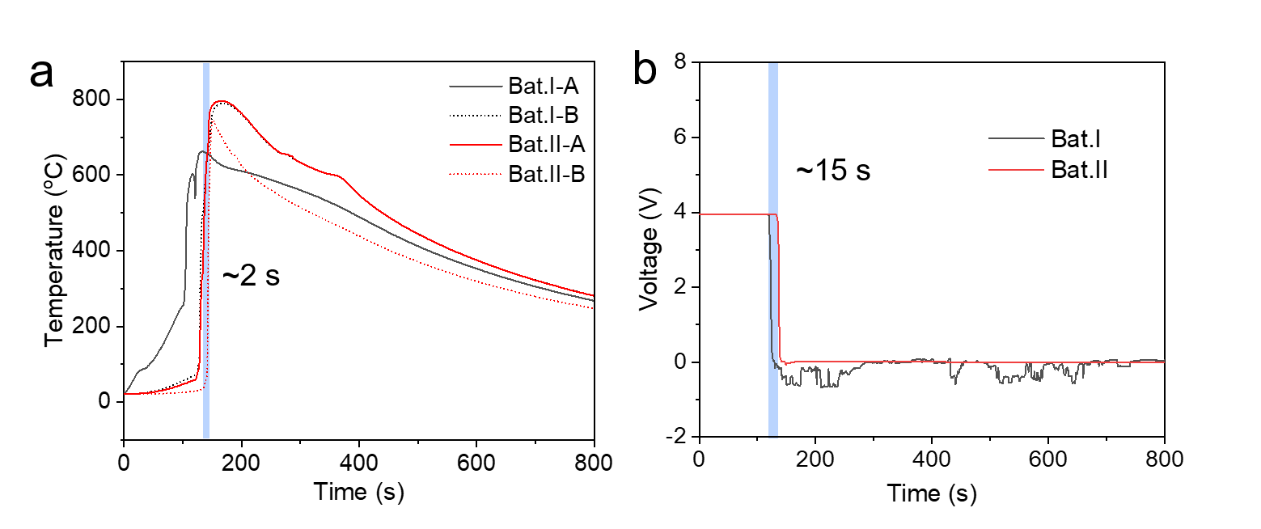


**Figure S20**. (a) Real-time temperature curves and (b) voltage of the Li-ion batteries without the protection of the RPS composite aerogel.

**Table S1**. Name and recipes of polyimide-silica aerogel dispersions

| Sample | Solid content of  polyimide solutions (wt%) | Weight of polyimide solutions (g) | Weight of silica aerogel (g) |
| --- | --- | --- | --- |
| RPS-20%-0 | 20% | 20 | 0 |
| RPS-20%-0.5 | 20% | 20 | 0.5 |
| RPS-20%-1.0 | 20% | 20 | 1 |
| RPS-15%-1.2 | 15% | 20 | 1.2 |
| RPS-15%-1.9 | 15% | 20 | 1.9 |
| RPS-10%-1.9 | 10% | 20 | 1.9 |

**Table S2**. Comparison of the polyimide-based aerogels in terms of thermal conductivity and compressional Young's modulus

| Sample | *ρ*  g/cm^3^ | *σ*_c_  MPa | *E*  MPa | *λ*  mW/m.K | Reference |
| --- | --- | --- | --- | --- | --- |
| Polyimide | 0.11 | 0.41 | 6.5 | 30.8 | [S5] |
|  | 0.087 | 0.3 | 3.2 | ~37.0 |  |
|  | 0.088 | 0.52 | 8.9 | 41.0 |  |
|  | 0.109 | 0.39 | 5.1 | 37.0 |  |
|  | 0.108 | 0.42 | 8.4 | 30.6 |  |
|  | 0.127 | 0.78 | 9.3 | 25.7 |  |
| Aramid nanofiber | 0.0047 | - | 0.014 | 29.2 | [S6] |
|  | 0.0063 | - | 0.027 | 32.5 |  |
|  | 0.0086 | - | 0.081 | 35.0 |  |
|  | 0.0129 | - | 0.159 | 40.0 |  |
| Cellulose | 0.023 | 0.076 | 0.0694 | 26.0 | [S7] |
|  | 0.035 | 0.17 | 1.005 | 28.0 |  |
|  | 0.046 | - | 3.657 | 29.0 |  |
|  | 0.055 | - | 2.052 | 37.0 |  |
|  | 0.064 | - | 1.797 | 45.0 |  |
|  | 0.072 | 1.26 | 1.116 | 49.0 |  |
| Alginate | 0.022 | 0.31 | ~0.40 | ~29.0 | [S8] |
|  | 0.032 | 0.85 | ~1.20 | ~28.5 |  |
|  | 0.042 | 1.75 | ~1.60 | ~28.4 |  |
|  | 0.053 | 2.1 | ~2.75 | 27.2 |  |
|  | 0.062 | 2.6 | ~3.26 | 31.3 |  |
| Silica | 0.077 | 2.1 | 0.25 | 16.3 | [S9] |
|  | 0.115 | 3.9 | 0.78 | 14.5 |  |
|  | 0.194 | 1.6 | 3.55 | 16.4 |  |
|  | 0.269 | 0.6 | 10.48 | 20.3 |  |
|  | 0.071 | 2.1 | 0.36 | 18.2 |  |
|  | 0.112 | 0.8 | 1.64 | 15.8 |  |
|  | 0.184 | 0.8 | 7.71 | 17.8 |  |
|  | 0.261 | 0.6 | 17.35 | 23.3 |  |
| Polyimide/silica | 0.196 | ~0.7 | ~9.00 | 20.8 | [S10] |
|  | 0.218 | ~0.9 | ~8.5 | 21.3 |  |
|  | 0.226 | ~1.1 | ~14.0 | 22.4 |  |
|  | 0.192 | ~1.0 | ~14.5 | 21.1 |  |
|  | 0.204 | ~0.75 | ~11 | 22.2 |  |
| Silk fibroin/silica | 0.11 | 2.50 | 3.10 | 32.0 | [S11] |
|  | 0.15 | 14.0 | 18.1 | 40.0 |  |
|  | 0.157 | 0.11 | 0.7 | ~36.0 |  |
|  | 0.232 | 0.10 | 0.8 | 43.0 |  |
| Polyorganosiloxane | 0.170 | - | 4.0 | 15.0 | [S12] |
|  | 0.18 | - | 7.8 | 15.3 |  |
|  | 0.22 | - | 4.7 | 15.3 |  |
|  | 0.190 | - | 3.3 | 15.2 |  |
|  | 0.21 | - | 7.7 | 15.4 |  |
|  | 0.230 | - | 5.6 | 16.4 |  |
| PVC | - | - | 0.007 | 120 | [S13] |
| PVC | - | - | 6.8 | 250 |  |
| PMMA | - | - | 118 | 180 |  |
| PC | - | - | 130 | 210 |  |
| PET |  |  | 80 | 300 |  |
| RPS-20%-0.5 | 0.458 | 8.0 | 235 _(initial modulus)_ | 41.3 | This work |
| RPS-20%-1.0 | 0.404 | 6.5 | 201_(initial modulus)_ | 38.1 |  |
| RPS-15%-1.9 | 0.232 | 2.2 | 20.5 | 23.7 |  |
| RPS-10%-1.5 | 0.217 | - | - | 21.6 |  |
| RPS-10%-1.9% | 0.183 | - | - | 20.4 |  |

ρ, bulk density; σ_c_, compress strength; E, Young’s modulus; λ, thermal conductivity.

**Table S3**. Comparison of RPS-20%-1.0 with the commercial fibers in terms of mechanical properties and decomposition temperature. Note that these values are from manufacturer websites and not the peer-reviewed literature.

| Products | Modulus  (MPa) | Decomposition temperature  (^o^C) | Density  （g/cm^3^） | Reference |
| --- | --- | --- | --- | --- |
| Kevlar® 29 | 70500 | 482 | 1.44 | [S14] |
| Kevlar® 49 | 112400 | 482 | 1.44 |  |
| Nylon 66 | 5517 | 254 | 1.16 |  |
| Polyester | 13793 | 256 | 1.38 |  |
| Polyethylene | 11724 | 149 | 0.97 |  |
| Fitlon® | 4260 | 521 | 1.41 |  |
| Hyplon® | 142000 | 550 (under N_2_) | 1.42 | [S15] |
| Suplon® | 6106 | 569 (under N_2_) | 1.41 |  |
| RPS-20%-1.0 | 201 | 583 | 0.40 | This work |

**Movie S1**. Additive manufacturing of the customized object for complex geometry demonstration.

**Movie S2**. Printed RPS-15%-1.9 honeycomb can maintain an intact morphology after withstanding an adult.

**Movie S3**. The printed RPS-15%-1.9 honeycomb shows good fireproof properties and can maintain its shape when exposed to a flame.

**Movie S4**. The fabric made of RPS-20%-1.0 and Kevlar can protect a chocolate figurine preserving its intact morphology when exposed to a burning propane torch.

References

[S1] M. J. Frisch, G. W. Trucks, H. B. Schlegel, G. E. Scuseria, M. A. Robb, J. R. Cheeseman, G. Scalmani, V. Barone, G. A. Petersson, H. Nakatsuji, X. Li, M. Caricato, A. V. Marenich, J. Bloino, B. G. Janesko, R. Gomperts, B. Mennucci, H. P. Hratchian, J. V. Ortiz, A. F. Izmaylov, J. L. Sonnenberg, D. Williams-Young, F. Ding, F. Lipparini, F. Egidi, J. Goings, B. Peng, A. Petrone, T. Henderson, D. Ranasinghe, V. G. Zakrzewski, J. Gao, N. Rega, G. Zheng, W. Liang, M. Hada, M. Ehara, K. Toyota, R. Fukuda, J. Hasegawa, M. Ishida, T. Nakajima, Y. Honda, O. Kitao, H. Nakai, T. Vreven, K. Throssell, J. A. Montgomery, Jr., J. E. Peralta, F. Ogliaro, M. J. Bearpark, J. J. Heyd, E. N. Brothers, K. N. Kudin, V. N. Staroverov, T. A. Keith, R. Kobayashi, J. Normand, K. Raghavachari, A. P. Rendell, J. C. Burant, S. S. Iyengar, J. Tomasi, M. Cossi, J. M. Millam, M. Klene, C. Adamo, R. Cammi, J. W. Ochterski, R. L. Martin, K. Morokuma, O. Farkas, J. B. Foresman, and D. J. Fox, Gaussian16, Revision C.01, Gaussian, Inc., Wallingford CT, **2019**.

[S2] T. Lu, F. Chen, Multiwfn: a multifunctional wavefunction analyzer. *J. Comput. Chem.* **2012**, *33*, 580.

[S3] L. A. Feldkamp, L. C. Davis, J. W. Kress, *J. Opt. Soc. Am. A*, **1984**, *1*, 612.

[S4]. D. Paganin, S. C. Mayo, T. E. Gureyev, P. R. Miller, S. W. Wilkins, *J. Microsc.* **2002**, *206*, 33.

[S5] Y. Sun, Z. Wang, J. Zhao, H. Nie, G. Zhou, *Chem. Eng. J.* **2023**, *479*, 147642.

[S6]. P. Hu, J. Wang, P. Zhang, F. Wu, Y. Cheng, J. Wang and Z. Sun, *Adv. Mater.* **2023**, *35*, e2207638.

[S7] M. Yan, Y. Pan, X. Cheng, Z. Zhang, Y. Deng, Z. Lun, L. Gong, M. Gao and H. Zhang, *ACS Appl. Mater. Interfaces* **2021**, *13*, 27458.

[S8] H. Xu, C. Liu, W. Guo, N. Li, Y. Chen, X. Meng, M. Zhai, S. Zhang and Z. Wang, *Chem. Eng. J.* **2024**, *489*, 151223.

[S9] S. Iswar, S. Galmarini, L. Bonanomi, J. Wernery, E. Roumeli, S. Nimalshantha, A. M. Ben Ishai, M. Lattuada, M. M. Koebel and W. J. Malfait, *Acta Mater.* **2021**, *213*, 116959.

[S10] L. Wang, J. Feng, S. Zhang, Q. Sun, Y. Luo, J. Men, W. He, Y. Jiang, L. Li, J. Feng, *Addit. Manuf.* **2023**, *71*, 103583.

[S11] H. Maleki, L. Whitmore, N. Husing, *J. Mater. Chem. A* **2018**, *6*, 12598.

[S12] G. Zu, K. Kanamori, T. Shimizu, Y. Zhu, A. Maeno, H. Kaji, K. Nakanishi, J. Shen, *Chem. Mater*. **2018**, *30*, 2759.

[S13] D. Häusermann, S. Bodry, F. Wiesemüller, A. Miriyev, S. Siegrist, F. Fu, S. Gaan, M. M. Koebel, W. J. Malfait, S. Zhao, M. Kovač, *Adv. Intell. Syst*. **2023**, *5*, 2300101.

[S14]https://www.dupont.cn/content/dam/dupont/amer/us/en/safety/public/documents/cn/kevlar-technical-user-guide-2010.pdf

[15] http://www.asxc.com.cn/gb2312/chanpinyuyingyong/chanpinzhongxin/
